# The Southern European Atlantic Diet and Its Supplements: The Chemical Bases of Its Anticancer Properties

**DOI:** 10.3390/nu15194274

**Published:** 2023-10-06

**Authors:** Pablo García Vivanco, Pablo Taboada, Alberto Coelho

**Affiliations:** 1 Spanish Academy of Nutrition and Dietetics, 31006 Pamplona, Spain; 2 Nutrition and Digestive Working Group, Spanish Society of Clinical, Family, and Community Pharmacy (SEFAC), 28045 Madrid, Spain; 3 Department of Condensed Matter Physics, Faculty of Physics, University of Santiago de Compostela, 15782 Santiago de Compostela, Spain; 4 Institute of Materials-USC (IMATUS), University of Santiago de Compostela, 15782 Santiago de Compostela, Spain; 5 Department of Organic Chemistry, Faculty of Pharmacy, University of Santiago de Compostela, 15782 Santiago de Compostela, Spain

**Keywords:** Southern Atlantic diet, anticancer, glucosinolates, sulforaphane, phenolic compounds, flavonoids, antioxidant, chemoprevention, nutragenomic, nutraceutical

## Abstract

Scientific evidence increasingly supports the strong link between diet and health, acknowledging that a well-balanced diet plays a crucial role in preventing chronic diseases such as obesity, diabetes, cardiovascular issues, and certain types of cancer. This perspective opens the door to developing precision diets, particularly tailored for individuals at risk of developing cancer. It encompasses a vast research area and involves the study of an expanding array of compounds with multilevel “omics” compositions, including genomics, transcriptomics, proteomics, epigenomics, miRNomics, and metabolomics. We review here the components of the Southern European Atlantic Diet (SEAD) from both a chemical and pharmacological standpoint. The information sources consulted, complemented by crystallographic data from the Protein Data Bank, establish a direct link between the SEAD and its anticancer properties. The data collected strongly suggest that SEAD offers an exceptionally healthy profile, particularly due to the presence of beneficial biomolecules in its foods. The inclusion of olive oil and paprika in this diet provides numerous health benefits, and scientific evidence supports the anticancer properties of dietary supplements with biomolecules sourced from vegetables of the brassica genus. Nonetheless, further research is warranted in this field to gain deeper insights into the potential benefits of the SEAD’s bioactive compounds against cancer.

## 1. Introduction

Cancer is a major global health concern, ranking as the second leading cause of death worldwide. The World Health Organization (WHO) emphasizes that modifying or avoiding key risk factors can prevent 30% to 50% of cancer-related deaths [1]. These factors include maintaining a healthy weight, adopting healthy habits, and, crucially, proper nutrition. Dietary and nutritional factors play a substantial role in cancer, with approximately one-third of cancer cases attributed to them, according to the International Agency for Research on Cancer (IARC) [2]. High consumption of ultra-processed foods, common in fast food, has been linked to increased risks of colon, breast, and prostate cancer [3]. Epigenetics, the study of gene expression changes unrelated to DNA sequence alterations, explores how external factors such as diet can activate or deactivate genes [4].

Emerging scientific evidence suggests that nutraceuticals, natural nutritional agents, can influence miRNA expression and cellular responses, including cancer prevention. The diet’s impact on DNA methylation, a gene-regulating chemical modification, underscores the importance of dietary biomolecules as antioxidants that safeguard against DNA damage and regulate DNA methylation [5,6].

**Figure 1:**
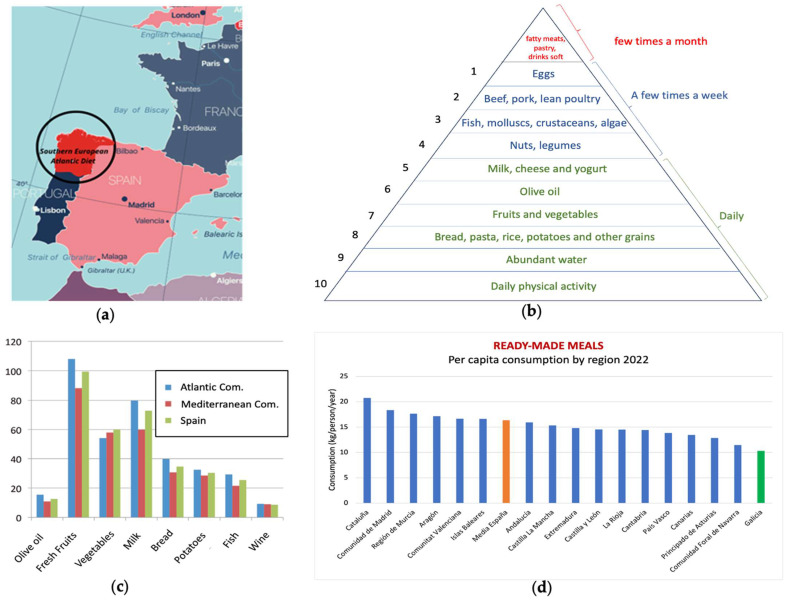
(**a**) Territorial zone covered by the SEAD; (**b**) nutritional pyramid of the South European Atlantic Diet, modified and adapted from [8]; (**c**) comparative consumption of the main beneficial elements of Iberian diets and the whole of Spain, adapted from [9]; (**d**) consumption of ready-made meals in Spain (by Regions, average of Spain in orange bar, Galicia in green bar): own elaboration. Source: Ministry of Agriculture, Fisheries, and Food, Government of Spain, Food consumption report of Spain, 2022, available at [14].

The Southern European Atlantic Diet (SEAD) [7,8,9,10], traditionally followed by populations along the South Atlantic coast, focuses on fresh local foods [11], especially from the Atlantic Ocean (Figure 1a). Three pillars define the SEAD: a phytoplankton-rich food chain yielding quality marine products such as fish, seafood, and seaweed [12]; distinctive inland products, notably brassica vegetables and abundant olive oil consumption; and culinary techniques that preserve raw material nutritional value, such as stewing and roasting.

Compared to the Mediterranean diet, the SEAD exhibits distinctions, emphasizing marine products, local vegetables, bread, dairy, and higher olive oil intake as shown in the nutritional pyramid and the key food consumption graph (Figure 1b,c) [13]. The Atlantic climate’s influence shapes culinary preferences and ingredient usage in SEAD dishes. Recent data from the Spanish Ministry of Agriculture, Fisheries, and Food highlight a preference for fresh seasonal foods, particularly in Galicia, with lower per-capita consumption of ready-made dishes (Figure 1d). Galician coasts, featuring “Galician rías” estuaries, provide unique conditions for high-quality seafood cultivation. These estuaries, created by river valleys flowing into the Atlantic Ocean, combine freshwater and saltwater, creating a nutrient-rich ecosystem conducive to diverse fish and shellfish.

SEAD includes a wide range of fresh seasonal vegetables, greens, and fruits. Notable vegetables such as turnip tops, cabbage, collard greens, potatoes, onions, and garlic are staples in traditional dishes, providing essential nutrients for digestive health and overall well-being.

The primary source of fats in the SEAD is olive oil, although sunflower or rapeseed oils are used in Portugal. Galicia boasts high-quality meats, leading to two Protected Geographical Indications (PGI): “PGI Ternera Gallega” (PGI Galician Veal), recognized by the European Union since 1996 [15], and “PGI Vaca y Buey de Galicia” (Galician Cow and Ox), with the most well-known native breed being the “Rubia Gallega” cow [16]. This meat is characterized by an intramuscular fat percentage exceeding 5.6% on average. Galicia also offers other quality meats, including a poultry PGI [17]. In Asturias, Asturian veal PGI [18], and in northern Portugal, other quality PGI meats can be found [19]. In the Bierzo area of Spain, another PGI is produced [20].

Shellfish aquaculture in Galicia relies on “bateas”, floating structures facilitating the cultivation of mussels, oysters, and scallops. Mussel cultivation is sustainable and known for its high quality and highly recognized and appreciated in the South European Atlantic Diet (SEAD).

The quality of the SEAD can be attributed to the trophic chain. A study by the CSIC determined the abundance and diversity of photosynthetic microorganisms in the Vigo Estuary, making it an autotrophic ecosystem [21]. Galicia’s marine location, daily sunlight, water currents, and temperature confluence create a highly fertile sea with abundant marine flora and fauna. This forms a food chain of great nutritional value, starting from phytoplankton, algae, crustaceans, and small fish, all the way up to large fish [22].

**Figure 2:**
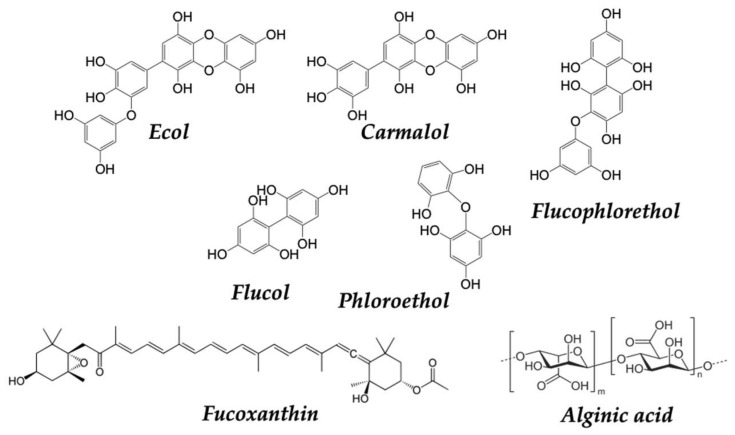
Active compounds found in brown algae native to the SEAD.

Algae play a fundamental role in this ecosystem. Galician algae are rich in antioxidants and anticancer properties, containing essential bioactive molecules such as fucoxanthin and phlorotannins (Figure 2). Fucoxanthin, a carotenoid, is abundant in “Kombu” algae, especially the Saccharina latissima, which grows in cold waters, along with *Undaria pinnatifida (Wakame)*, characteristic of the Galician coasts [23]. At the molecular level, fucoxanthin is a xanthophyll [24], which is structurally similar to beta-carotene and vitamin A and has promising health benefits. Algae also contain alginic acid, enriching the trophic chain. While not a traditional dish in this region, algae are becoming increasingly incorporated into the SEAD. Algae biotechnology [25] is growing in applications for pharmaceuticals, cosmetics, renewable energy, and feed for fish, mollusks, and crustaceans. Algae are gaining attention in nutrition due to their fiber content and low caloric value, aligning with Galicia’s “Zero Obesity 2022–2030” initiative [26].

The GALIAT6 + 7 project, driven by six companies and seven scientific groups, conducted a randomized, family-focused dietary intervention to evaluate the Atlantic diet’s quality. This diet has been linked to health and longevity benefits [27,28]. People following this dietary pattern have a lower risk of cardiovascular diseases, type 2 diabetes, obesity, and certain types of cancer [28]. At the epigenetic level, the SEAD has shown positive effects, modulating DNA methylation and histone modification, impacting the regulation of genes related to health and longevity [13]. As a result of this epigenetics, Galicia is beginning to receive worldwide attention for its longevity, and there are several professionals from different disciplines, entrepreneurs and associations that study this phenomenon of centenarians in Ourense [29], where there are 384 centenarians, equivalent to 126 per 100,000 inhabitants, or Lugo with 333, which is equivalent to 96 per 100,000 inhabitants [30], comparable to longevity paradigms such as Icaria in Greece or Okinawa in Japan [31,32].

An important study was conducted in recent years by Carballo-Casla and coworkers, related to the properties of the SEAD and all-cause mortality in older adults [33]. Another study confirmed that the SEAD is associated with lower concentrations of markers of coronary risk [34]. Adherence to the SEAD and occurrence of non-fatal acute myocardial infarction were analyzed by Oliveira and coworkers [35], and Tejera-Pérez reported a study about the SEAD and a recent proposal of the SEAD Index [7]. They concluded that SEAD should be considered an excellent dietary pattern and lifestyle. In addition, a study about the SEAD and depression risk in Europe was also reported [33]. Romero-Rodríguez and coworkers performed a thirteen-item questionnaire developed to assess the degree of adherence to the SEAD pattern of the Galician senior population. Another questionnaire was designed to determine the degree of knowledge about the SEAD. The results confirmed that the adherence to this diet was medium to high [36].

In this review, which focuses more on Galicia and northern Portugal regions, our aim is to highlight the main components and biomolecules present in the SEAD, which, according to data, contribute to longevity in the Galician–Portuguese population [33,34,35]. The main objective of this work is, therefore, to compile and analyze for the first time bioactive compounds with anticancer properties found in significant amounts and consumption in the components of the SEAD foods from a chemical and pharmacological perspective as well as their specificities in relation to other diets worldwide.

## 2. Discussion

For the study of the gathered information, the following databases were consulted: Scifinder, PubMed, Protein Data Bank (PDB), and the Galiat6 + 7 [37]. Additionally, other government sources at the national or regional level were utilized.

The analysis of the information, which encompasses different perspectives (dietary-nutritional, chemical, and pharmacological), has been carried out considering each representative food of the diet that, due to its frequent consumption in the areas of mainly Galicia, Asturias, and Northern Portugal, can be considered intrinsic to the Atlantic diet. From each food, the main components that have shown scientific evidence of possessing antioxidant, antiproliferative, and overall anticancer activities have been selected.

### 2.1. Epigenetics and Cancer—Longevity and Benefits of the Atlantic Diet—Previous Studies

In recent decades, there has been a significant increase in cancer cases worldwide. While various factors may contribute to this rise, it has been shown that changes in dietary habits play a significant role in cancer development and progression. Epigenetic events are accepted as the most important mechanisms in the development and progression of cancer [38,39]. The connection between diet and cancer is partly due to the effects of diet on epigenetics. However, it is important to note that the field of epigenetics and its relationship to diet and cancer are still evolving, and further research is needed to fully understand the mechanisms and clinical implications. Collectively, several epigenetic events can be triggered by the diet, some of which include

DNA methylation: Methylation is a process in which a methyl group is added to DNA, which can silence gene expression. Diet can influence DNA methylation, affecting gene regulation and contributing to cancer development [40]. Cancer cells are characterized by genomic instability associated with hypomethylation in Cytosine Phosphorus Guanine (CpG) in intergenic regions and repetitive elements, but also by marked hypermethylation of CpG islands in tumor-suppressor genes. Several studies have indicated that some miRNA genes reside near CpG islands, so their expression can be regulated by epigenetic modifications at these sites [41,42].

DNA methylation or methylation of cytosine in the context of the CpG dinucleotide has important consequences on our genome. When hypermethylation occurs in a tumor-suppressor gene promoter, it silences the gene and allows tumor cells to grow in a manner similar to deletions or mutations [43]. DNA methylation errors, in terms of hypermethylation or hypomethylation, are critically involved in tumorigenesis [44].

Histone modification: Histones are proteins that help package DNA into a compact structure. Histone modifications, such as acetylation and methylation, can regulate gene expression. Diet can affect histone modifications, which can in turn influence cancer risk [45].

MicroRNAs: MicroRNAs are small RNA molecules that regulate gene expression. Diet can affect the expression of microRNAs, which can in turn influence the onset and progression of cancer. For instance, in relation to prostate cancer, among dietary components, polyphenols play a protective role either due to their antiaging and antioxidant properties or their more general “cell protection” effects [46].

There are numerous studies that have investigated the relationship between changes in dietary habits and epigenetic events in humans. It has been observed that certain nutrients and bioactive compounds present in foods, such as polyphenols, specific vitamins, and omega-3 fatty acids, can have epigenetic effects and modify cancer risk [47,48]. A recent review analyzes the effect of diet components on cancer with epigenetic mechanisms. This study focused on the analysis of bioactive dietary components such as resveratrol, genistein, quercetin, curcumin, isothiocyanates (sulforaphane in broccoli sprouts), tea polyphenols, selenium, withaferin A, indole-3-carbinol, and their role in reducing cancer risk [49].

Cellular deterioration or disruption of homeostasis is often related to oxidative stress caused by free radicals or reactive oxygen species (ROS). Free radicals are unstable molecules that are produced in the body, as a result of natural processes and external factors such as exposure to ultraviolet radiation, environmental pollution, tobacco consumption, dietary factors, among many others. These free radicals can cause damage to DNA and other cellular molecules leading to mutations and epigenetic alterations that contribute to cancer development [50].

Antioxidants act as defenders against free radicals, neutralizing and preventing them from causing damage to cells [51]. By protecting DNA and other cellular molecules, antioxidants help prevent the occurrence of mutations and epigenetic changes [52]. These epigenetic modifications play a crucial role in gene activation or deactivation, and antioxidants can help maintain a proper balance in these processes which can have important implications in cancer prevention and treatment [53]. There are non-enzymatic compounds with a more pronounced secondary action, among which are vitamin C and E, carotenoids, flavonoids, phenols, polyphenols, phytoestrogens, selenium, lipoic acid, sulforaphanes, glucosinolates, and coenzyme Q10. In the SEAD, many foods are sources of these antioxidants and are consumed with interesting frequency.

In 2016, a group of researchers from the Department of Laboratory Medicine and the Department of Pediatrics at the University Hospital of Santiago de Compostela, Spain, initiated the GALIAT6 + 7 project. The main goal of this project was to examine the scientific evidence that could support the promotion of the Atlantic diet as a healthy choice, allowing its incorporation into preventive family health strategies in line with the cultural and gastronomic heritage of Europe’s Atlantic regions. The conclusion of the study was that adherence to the Atlantic diet has been associated with a decrease in body mass index (BMI) and an improvement in lipid profile, as revealed by the data from the GALIAT6 + 7 clinical trial. The GALIAT study was the first clinical trial to investigate the effects of the Atlantic diet on metabolic and cardiovascular health as well as adiposity. If the study hypothesis of this work is confirmed, this dietary pattern could be included in health promotion strategies.

A more recent review by Lorenzo analyzes the epigenetic effects of healthy foods and lifestyle habits from the SEAD pattern [13]. The review evaluates the key aspects that define the SEAD and the potential epigenetic changes associated with its components, based on recent studies [13,27].

### 2.2. Nature of the Components of the Southern European Atlantic Diet (SEAD)

Many fruits and vegetables contain natural compounds that act as blocking or neutralizing agents against carcinogens. These compounds can contribute to cancer protection by preventing the formation of cancer cells or inhibiting their growth.

Phenols: Phenols are antioxidant compounds found in various fruits and vegetables. Phenols neutralize free radicals and protect cells against oxidative damage, which can reduce the risk of cancer. Phenolic compounds comprise three groups: simple phenols (e.g., tyrosol, hydroxytyrosol, or 3,4-dihydroxyphenylethanol), phenolic acids (e.g., caffeic acid), and flavonoids (e.g., quercetin) [54].

Indoles: Indoles are compounds present in cruciferous vegetables such as broccoli, kale, and cauliflower. Indoles have demonstrated anticancer properties by modulating the activity of certain enzymes responsible for detoxifying carcinogens and interfering with cell growth-related signaling pathways. The biological role of I3C (indole-3-carbinol) and DIM (3,3’-diindolylmethane) is particularly noteworthy [55].

Flavones: Flavones are a type of flavonoid found in citrus fruits, such as oranges and lemons, as well as certain vegetables and herbs. These compounds have antioxidant and anti-inflammatory properties, and have been shown to inhibit the proliferation of cancer cells and promote apoptosis (programmed cell death) [56,57].

Isothiocyanates: Isothiocyanates are compounds formed from glucosinolates present in cruciferous vegetables, such as broccoli, horseradish, and Brussels sprouts. Isothiocyanates have demonstrated anticancer effects by inhibiting the proliferation of cancer cells, inducing apoptosis, and blocking tumor formation.

All these compounds can protect in various ways:

Antioxidant action: Many of these compounds have antioxidant properties, which means they can neutralize free radicals and other reactive oxygen compounds that can damage DNA and cells. By reducing oxidative stress, these compounds can prevent cellular damage and reduce the risk of mutations that could lead to cancer [58,59].

Anti-inflammatory activity: Some of these blocking agents have anti-inflammatory properties, which may be important in cancer prevention. Chronic inflammation has been associated with an increased risk of cancer, and compounds with anti-inflammatory properties can help reduce inflammation and, therefore, decrease the risk of cancer cell development [60].

Modulation of detoxifying enzymes: Some of these compounds can modulate the activity of detoxifying enzymes in the body, such as phase II enzymes, which are involved in the elimination of carcinogens and other toxic compounds from the body. By increasing the activity of these enzymes, blocking compounds can help eliminate carcinogens before they can damage cells [61].

Influence on gene expression: Some of these compounds can affect the expression of genes related to cell proliferation, apoptosis (programmed cell death), and cellular signaling. These effects can help prevent tumor formation and growth [62].

Regulation of hormonal metabolism: Some blocking agents can influence hormonal metabolism and help balance the levels of hormones related to cancer development, such as estrogens. This can be especially relevant in the case of certain hormone-related cancers, such as breast cancer [63].

### 2.3. Analysis of the Foods and Key Components in the Anticancer Activity of the SEAD

#### 2.3.1. Mostly Consumed Fish and Seafood in the SEAD: Mussels, Octopus and Cod

Spain is the second-largest producer of mussels (Mytilus galloprovincialis) in the world, and almost all of it is produced in Galicia. The quality of Galician mussels is extraordinary, and it has been recognized by the European Commission with the “Protected Designation of Origin “POD Mejillón de Galicia” [64] as a mark of quality.

**Figure 3:**
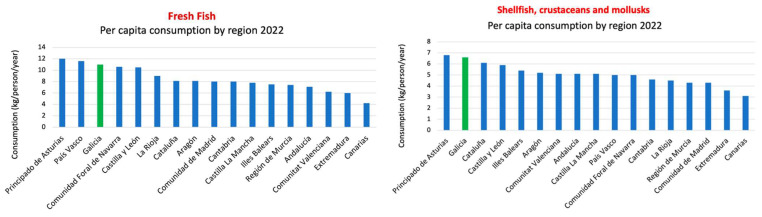
Fresh fish, shellfish, crustaceans and mollusks consumption in Spain (2022), Galicia in green bar. Self-made. Source: Ministry of Agriculture, Fisheries and Food, Government of Spain [14].

The consumption of fresh fish is high, with Asturias ranking as the first and Galicia the third autonomous community in Spain in terms of per-capita consumption. As for seafood consumption in Galicia, according to the Food Consumption Report of Spain 2022, it is also very high, placing the region in the second position (Figure 3). Among these highly consumed food groups in the SEAD, we will focus on three of them due to their high consumption and their content of anticancer substances. Mussels are rich in various components that are currently being investigated, among which betaines and selenium stand out.

**Figure 4:**
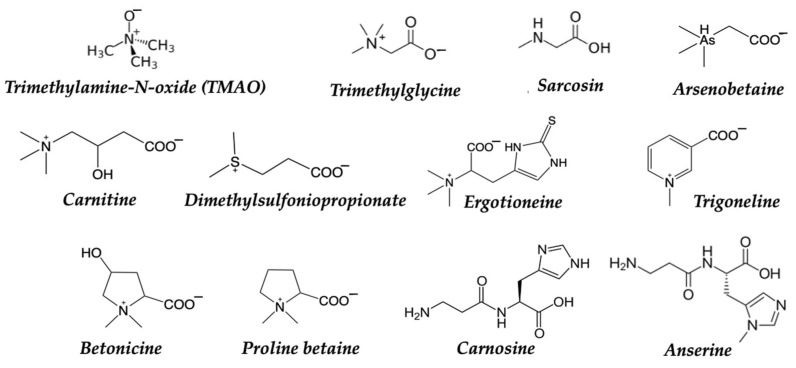
Main betaines in the diet and other biomolecules present in mussels and octopus.

Betaines: They are chemical compounds formed by a methyl group and an amino group (Figure 4) found in a variety of foods, such as beets, spinach, whole grains, and seafood. Betaine exists widely in animals and plants. Among plants, Lycium barbarum and legumes contain betaine. Sugar beet molasses is the main source of betaine. Among animals, the liver, spleen, and amniotic fluid of mollusks such as octopus, cuttlefish, and shrimp, as well as vertebrates (including humans), contain betaine. These compounds have been studied in relation to cancer and their potential effects. Some studies have explored the role of betaines in the prevention and treatment of cancer. It has been observed that betaines can have anticancer properties because they are believed to act as methyl group donors [65]. This means that they can transfer methyl groups to different molecules in the body, which can have beneficial effects on various metabolic pathways. For example, it has been observed that betaines can participate in DNA methylation and other molecules, which can regulate gene expression and other cellular processes [66].

The effects of trimethylglycine (TMG) on colorectal cancer cell lines have been investigated. The results showed that TMG inhibited cell proliferation and promoted apoptosis in cancer cells, suggesting a potential antitumor effect. The effects of TMG on breast cancer models were also examined. The results indicated that TMG reduced cell migration and invasion, in addition to inhibiting tumor growth in mice. In the case of mussels, the presence of different types of betaines has been identified (Figure 4), with trimethylglycine (TMG) being the most common [60]. Other related compounds, such as dimethylglycine (DMG) and sarcosine, have also been detected.

The betaines present in mussels have antioxidant properties, anti-inflammatory effects, and promote healthy cardiovascular function. Anserine is a dipeptide that contains betaine and is found in the muscle tissue of mussels, along with other amino acids. It has antioxidant properties and has been investigated for its potential involvement in muscle health [67]. Carnosine is a similar dipeptide (formed by the combination of the amino acids beta-alanine and L-histidine) that is found in various tissues and foods, including muscle and some meat products such as octopus. The anticancer effects of Carnosine have been described. It inhibits breast, ovarian, colon, and leukemic cancer cell proliferation; upregulates the expression of pro-inflammatory molecules; modulates cytokine secretion and alters U937 differentiation and phenotype. These effects may have implications for a role for Carnosine in anticancer therapy [68].

**Scheme 1:**
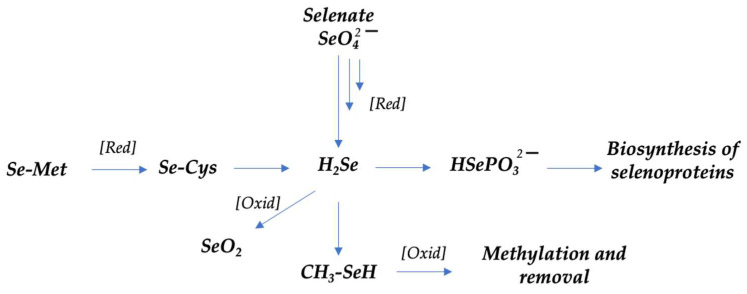
Main metabolic reactions of organic and inorganic Se compounds in humans. Adapted and modified from [71]. Briefly, H_2_Se is formed during reduction both inorganic (selenate) and organic (SeMet), species. Both SeMet and CH_3_SeCys are enzymatically converted to CH_3_SeH. Glutathione (GSH) or other thiols undergo these reactions when a reduction in inorganic Se species occurs. Reduction–oxidation species are generated by two presented reactions. SeMet—L-selenomethionine, SeCys—selenocysteine, CH_3_SeH—monomethylselenol, H_2_Se—hydrogen selenide, HSePO_3_—selenophosphate, and SeO_2_—Se dioxide.

Selenium: The optimal dietary level of selenium ensures its proper antioxidant and anticancer activity. Special attention is due to the antioxidant activities of selenium compounds, especially selenoproteins [69], and their importance in antioxidant defense. It is worth noting that data on selenium’s anticancer properties are still controversial. Moreover, selenium compounds as chemotherapeutic agents are usually used at supranutritional doses [70]. The main metabolic reactions of organic and inorganic selenium compounds in humans [71,72] are shown in Scheme 1.

Galician octopus (*Octopus vulgaris*) is the main ingredient of one of the prominent dishes of the SEAD, “pulpo a la gallega” or also known as “pulpo a la feria.” This festive dish is prepared with whole cooked octopus (usually in copper pots) and is a common presence at festivals, fairs, and pilgrimages in Galicia. It is served on a wooden plate and is often accompanied by the famous “cachelos,” skin-on boiled potatoes, and generously drizzled with olive oil and sweet or spicy paprika. A significant percentage of the octopus consumed in Galicia, which is part of the SEAD, is not native to the region, and the majority comes from Morocco [73], a country that exports various seafood products to Spain.

Octopus is a source of several bioactive compounds that may have beneficial health properties. Some of the main bioactive compounds present in octopus are as follows:

Omega-3 and polyunsaturated fatty acids: Octopus is a source of omega-3 fatty acids, which have been associated with various health benefits, including protection against cancer. These fatty acids have anti-inflammatory properties and may influence cellular growth and proliferation mechanisms.

Taurine: It is an amino acid found in high concentrations in octopus. Taurine has been suggested to have antioxidant properties and may play a role in cancer prevention [74]. Recently, taurine not only mitigates the side effects of chemotherapy in cancer but also possesses antitumor properties, including inhibiting cancer cell proliferation and inducing apoptosis in certain cancers by differentially regulating proapoptotic and antiapoptotic proteins. The underlying molecular mechanism also suggested that taurine can be a potential clinical application in tumor therapy [75].

Polysaccharides: Octopuses and other cephalopods contain various types of polysaccharides, such as chitin and glucans. Some studies have investigated the immunomodulatory and antitumor properties of marine-origin polysaccharides, including those found in octopuses. Particularly, Glycosaminoglycans (GAGs) are an important component of the tumor microenvironment (TME) [76]. It has been observed that certain marine polysaccharides can stimulate the immune system and enhance the activity of immune cells, such as macrophages and lymphocytes, which could have implications in the response against cancer. In in vitro and animal studies, some marine-origin polysaccharides have demonstrated antitumor activity by inhibiting the growth of cancer cells, inducing apoptosis, and reducing angiogenesis (formation of new blood vessels in the tumor). Some studies have confirmed interesting antiproliferative effects of glycosaminoglycans present in the Norwegian lobster [77] and sea squid [78]. More research is needed in this field.

Bioactive peptides: Some octopus species produce toxic secretions as a predatory and defense mechanism in specialized organs called posterior secretory glands. The bioactive substances in these secretions can be small molecules such as histidine or peptides such as tachykinins. Octopus venoms contain tachykinin peptides that, despite being isolated from an invertebrate, exhibit characteristics similar as vertebrate peptides. The actions of tachykinin peptides are mediated by one or more tachykinin receptors, which, in the case of vertebrates, are neurokinin 1 receptor (NK1R), neurokinin 2 receptor (NK2R), and neurokinin 3 receptor (NK3R). It was shown that the most potent form of tachykinin, Oct-TK-III, was not only the most anionically charged but also the most structurally stable [79,80].

**Figure 5:**
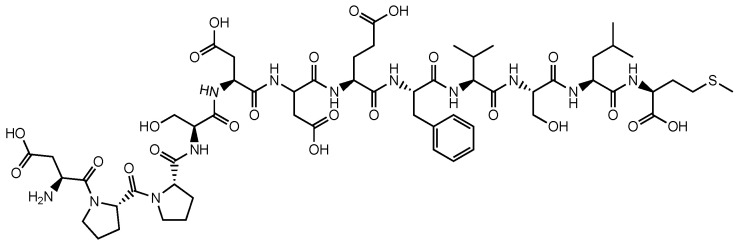
Chemical structure of OCTPEP-1 peptide.

In *Octopus vulgaris*, the taquikinins Oct-TK-I and Oct-TK-II are found, which are also active in vertebrates. However, a comparison of their relative effects in vertebrate and invertebrate tissues has not been conducted. Taquikinins are involved in central nervous system pathways that mediate pain, anxiety, motor coordination, and cognition. Therefore, the taquikinins in the venom of *Octopus vulgaris* could be beneficial for the treatment of various disorders, such as irritable bowel syndrome, asthma, chronic pain, depression, Parkinson’s disease, breast cancer, and lung cancer [79,81]. In other recent studies, such as that conducted by Maria P. Ikonomopoulou et al., a peptide isolated from and modified in the Australian species *Octopus kaurna* (Figure 5), known as Octpep-1, was investigated [82,83]. They studied its anticancer profile and its potential as a drug against melanoma with mutation in the BRAF gene, the dominant form of this disease, and with minimal effect on fibroblasts. Melanoma is the main skin cancer and causes about 57,000 deaths worldwide. The Octpep-1 peptide found in the ink reduced tumor progression in melanoma xenografts in mice and zebrafish. Therefore, it mediates selective cytotoxicity in BRAF-mutated melanoma in vitro and prevents tumor progression in vivo, providing a basis for melanoma therapy [83].

During the last decade, several studies related to octopus extracts and their potential antiproliferative activity have been conducted. A study conducted by Carolina Moreno-Félix et al. in 2012 examined fractions from an organic extract of fresh octopus. (*Paraoctopus limaculatus*) for biological activities such as antimutagenic and antiproliferative properties using Salmonella tester strains TA98 and TA100 with metabolic activation. This study indicated that within several compounds in the lipid fraction of the octopus, a group of saturated and unsaturated fatty acids are responsible for the bioactivity against AFB1 mutagenicity or proliferation of murine cancer cells, or both. The isolation and identification of the actual antimutagenic and antiproliferative compounds in octopus are the focus of ongoing research [84].

In another study by Finaia and coworkers, it was shown for the first time that lipid extracts from octopus by-products (*Octopus vulgaris*) have antiproliferative and apoptotic effects on human breast cancer cell lines [85].

Martín S. Hernandez Zazueta et al. investigated the antiproliferative and anti-inflammatory effects of *Octopus vulgaris* ink extracts (hexane, ethyl acetate, dichloromethane and water extracts) in human colorectal cancer cells (HT-29/HCT116) and breast cancer cells (MDA-MB-231), as well as RAW 264.7 murine cells treated with Lipopolysaccharide (LPS). All extracts, except ethyl acetate, exhibited antiproliferative effects without being cytotoxic to healthy ARPE-19 and RAW 264.7 cells [86]. Furthermore, from the *Octopus vulgaris* ink extracts, an antiproliferative and proapoptotic activity was observed for a metabolite called N-(2-ozoazepa-n3-yl)-pyrrolidine-2-carboxamide, known as Ozopromide (OPC). This novel metabolite derived from *Octopus vulgaris* ink demonstrates proapoptotic effects on A549 lung cancer cells and inhibits pro-inflammatory markers [87]. Specifically, antiproliferative activity has been also observed in cell lines of certain cancer types such as prostate (22Rv1), lung (A549), and cervical epithelioid adenocarcinoma (HeLa) [88]. Additionally, *Octopus vulgaris* extracts containing OPC have shown anti-inflammatory activity in LPS-stimulated RAW 264.7 cells, highlighting the potential of *Octopus vulgaris* ink as a nutraceutical product or supplement with antitumoral action [87].

**Figure 6:**
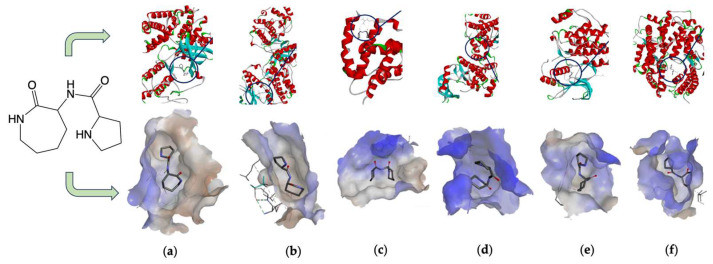
In silico analysis was performed using the AutoDock program to explore the most promising interactions between N-(2-oxoazepan-3-yl)-pyrrolidine-2-carboxamide (OPC) and selected cancer targets in 22Rv1 cells. The molecule of ozopromide was docked into the active sites of: (**a**) cyclin D1, (**b**) ERK1, (**c**) BAD, (**d**) c-SRC, (**e**) ERK, and (**f**) ACE2. Adapted and modified from [88].

The OPC also exhibited antioxidant and anti-inflammatory activity by reducing the production of reactive oxygen species (ROS) and affecting proinflammatory cytokines, as confirmed by in silico studies (Figure 6), demonstrating moderate affinity between OPC and these markers. In silico ADMET prediction studies suggested a lower potential for acute toxicity for OPC after oral administration, although further studies are needed to confirm this. Continued research is necessary to investigate the effects of OPC in in vitro and in vivo models of cancer and inflammation [87].

Cod (*Gadus morhua*) is a characteristic fish in the gastronomy of the SEAD, particularly in the northern region of Portugal. It is rich in nutrients and can provide several health-beneficial components. Although a direct relationship between cod components and anticancer activity has not been established, it is important to note that a diet rich in fish, such as cod, has been associated with overall health benefits, including the prevention of certain types of cancer.

Cod liver oil is also rich in vitamin D. One teaspoon (5 mL) of cod liver oil provides 15 μg (600 IU) of vitamin D3, a recommended daily amount. A recent meta-analysis confirms the anticancer properties of vitamin D, as numerous findings indicate that diet components, including vitamin D, may exert chemopreventive effects through alterations in microRNA (miRNA) expression [89].

Some of the beneficial components present in cod include:

Omega-3 fatty acids: Cod is an excellent source of omega-3 fatty acids, especially eicosapentaenoic acid (EPA) and docosahexaenoic acid (DHA). These fatty acids have demonstrated anti-inflammatory effects and may help reduce the risk of developing certain types of cancer.

High-quality proteins: Cod is a source of high-quality proteins that provide the essential amino acids necessary for proper cellular function and repair.

Vitamins and minerals: Cod contains a variety of essential vitamins and minerals, such as vitamin D, vitamin B12, vitamin A, iodine, selenium, and phosphorus. These nutrients are important for maintaining a healthy immune system and proper cellular function.

#### 2.3.2. Genuine Vegetables of the SEAD: Galician turnip Top, Garlic, Potato, and Padrón Pepper

**Figure 7:**
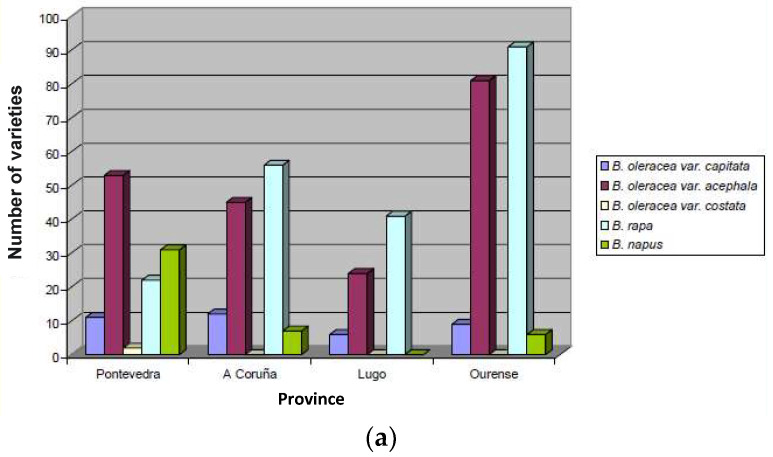

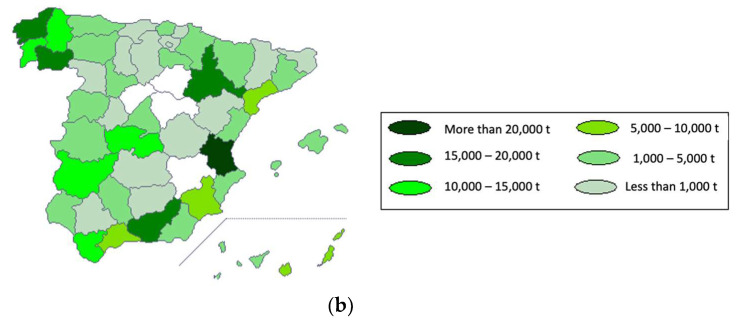
(**a**) Distribution of *Brassica* varieties by provinces in Galicia (up). (**b**) *Brassica oleracea* (cabbage) in Spain (down) production (in tonnes), adapted and modified from [17].

The Brassicaceae family, also known as the cruciferous family, comprises the most consumed vegetables within the SEAD, with Galicia standing out for its consumption compared to the rest of Spain. Some of the well-known plants and vegetables belonging to this family include cabbage (Brassica oleracea var. capitata), kale (Brassica oleracea var. sabellica), broccoli (Brassica oleracea var. italica), cauliflower (Brassica oleracea var. botrytis), Brussels sprouts (Brassica oleracea var. gemmifera), radish (Raphanus sativus), arugula (Eruca sativa), mustard (Brassica juncea, Brassica nigra), turnip (Brassica rapa), watercress (Nasturtium officinale), horseradish (Armoracia rusticana), Chinese cabbage (Brassica rapa var. pekinensis), and red-leaf kale (Brassica oleracea var. acephala). Turnip tops from Galicia are a food with a quality seal, the “PGI Grelos de Galicia” [90]. In the Portuguese part of the SEAD, another important vegetable from the Brassicaceae family is green kale. It is widely used in one of Portugal’s most typical dishes, “caldo verde,” where it is cut into strips and also used as a garnish. In Galicia, specifically, three species of the Brassica genus are cultivated, B. oleracea, B. napus, and B. rapa, for horticultural and fodder consumption (Figure 7) [91].

Each of the Brassica varieties has unique characteristics and nutritional benefits, but they all share certain similarities in their flower and fruit structures.

**Figure 8:**
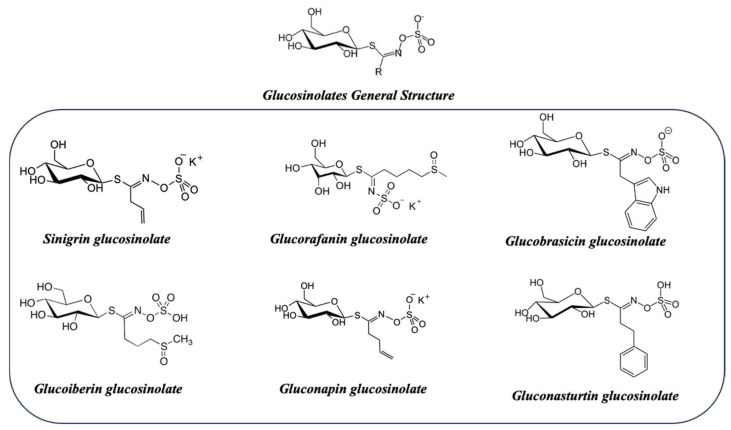
General structure of glucosinolates and main glucosinolates present in the SEAD.

Turnip top (Brassica rapa subsp. rapa), also known as Galician turnip top, is a leafy green vegetable that belongs to the species Brassica rapa, which is part of the Brassicaceae family [92]. Turnip top is a commonly cultivated and consumed plant in the Galicia region of Spain, closely integrated into the SEAD, where it is used in traditional dishes such as caldo gallego, cocido gallego, and lacón con grelos (Galician stewed pork with turnip tops). Turnip top leaves are rich in nutrients and have a bitter and spicy taste. As part of the Brassicaceae family, turnip top contains glucosinolates and sulforaphanes, phytochemical compounds with beneficial health properties. Glucosinolates are natural compounds found in plants of the Brassicaceae family and break down into biologically active compounds, such as sulforaphanes, when cut, chewed, or processed. Glucosinolates present in turnip top include sinigrin, glucoraphanin, glucobrassicin, and glucoiberin (Figure 8).

Glucoraphanin: One of the most common glucosinolates found in vegetables such as broccoli, Brussels sprouts, and kale. It is converted to sulforaphane, a bioactive compound with antioxidant properties and potential anticancer activity.

Sinigrin: Found in vegetables such as horseradish, mustard, and mustard seeds. It can be converted to allyl isothiocyanate, which has been associated with cardiovascular health benefits and antimicrobial properties.

Glucobrassicin: Found in vegetables such as cauliflower, cabbage, and Brussels sprouts. It can be converted to indole-3-carbinol, which has been studied for its potential to regulate hormonal metabolism and exhibit anticancer effects.

Gluconasturtiin: Found in vegetables such as watercress and arugula. It can be converted to phenylethyl isothiocyanate, which has shown antimicrobial and anti-inflammatory activity.

Glucoiberin: It is found particularly in the seeds and leaves of these plants.

Glucoraphanin: This is the most abundant glucosinolate in Galician turnip tops [37,93].

**Scheme 2:**
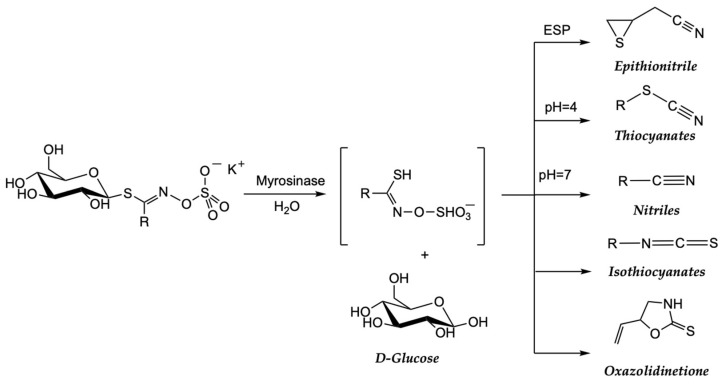
Hydrolysis products from glucosinolates.

All these compounds are converted into sulforaphanes through the action of the enzyme “myrosinase” when the plant cells are ruptured. Sulforaphanes are known for their antioxidant, anti-inflammatory, and potentially anticancer properties. The conversion process from glucosinolate to sulforaphane involves a series of biochemical steps:

In the plant, glucosinolates are stored in separate cellular compartments from the “myrosinase.” When plant tissues are damaged, such as when vegetables are cut or chewed, the myrosinase enzyme reacts with the glucosinolates. This interaction triggers a biochemical reaction known as hydrolysis, where myrosinase breaks the glucosidic bond of the glucosinolate and releases different products. It is important to consider that the process of converting glucosinolate to sulforaphane can be influenced by various factors, such as the plant variety, processing, and cooking. Therefore, when damage occurs in the plant, glucosinolates are degraded into a variety of hydrolytic products (catalyzed by myrosinase) that are responsible for almost all the biological activities of this class of compounds (Scheme 2). The process begins with the hydrolysis of the thioglucoside bond, resulting in the formation of glucose and an unstable aglucone.

In particular, one of the products released by the hydrolysis of glucosinolate is a compound called thioglucosinolate, which quickly reacts with a water molecule and forms an isothiocyanate, which is an active form of sulfur compound. Sulforaphane is an example of an isothiocyanate formed from certain glucosinolates present in vegetables such as broccoli, turnip tops and arugula.

The resulting sulforaphane from the conversion of glucosinolate to isothiocyanate has various beneficial biological activities. Sulforaphane has been shown to have antioxidant, anti-inflammatory, and anticancer properties [94], through the activity of phenethyl isothiocyanate, inhibiting the Signal Transducer and Activator of Transcription 3 (STAT3) activation in prostate cancer cells [95]. Another study analyzed the antioxidant activity of turnip top [92]. Furthermore, it can influence the expression of genes related to detoxification and cellular protection. Therefore, due to its glucosinolate content, turnip top is a product comparable and equivalent to broccoli.

**Scheme 3:**
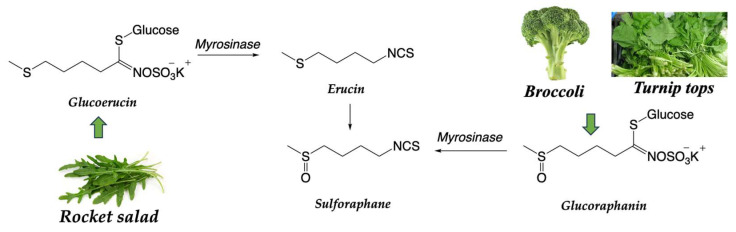
Conversion of glucosinolates found in rocket salad, turnip greens, or cruciferous vegetables into sulforaphane.

The most common sulforaphane in vegetables of the Brassicaceae family, such as broccoli, Brussels sprouts, and cabbages, is 1-isothiocyanate-4-(methylsulfinyl)butane, (4-MSOB). The precursor of sulforaphane is found in the form of a glucosinolate known as glucoraphanin. When *Brassicaceae* vegetables are cut, chewed, or processed, the myrosinase enzyme present in them converts glucoraphanin into sulforaphane, which is the active compound. Other source of sulforaphane is the glucoerucin from the rocket salad, which is converted in erucin by the myrosinase and finally reduce to give sulforaphane (Scheme 3).

The quantity and specific composition of glucosinolates and sulforaphanes can vary depending on factors such as the plant variety, cultivation, processing, and food preparation. The highest content of total glucosinolates is found in turnip tops and turnip greens [96]. Additionally, the content of glucosinolates and sulforaphanes may decrease with cooking time and temperature during cooking, as the myrosinase enzyme can be inactivated by heat, which is why it is recommended to cook them “al dente”.

Regarding the qualitative and quantitative analysis of these components in Galician cabbages and turnip tops, a study conducted by the Galician company Rosaleira [37], as part of the GALIAT6 + 7 project, focused on the study of functional biomolecules in Galician food products through agro-biotechnological research, and yielded the following conclusions:

Both fresh useful parts and fresh residue presented identical profiles of glucosinolates in both turnip tops and cabbages. In turnip tops, aliphatic glucosinolates were the major compounds, with gluconapin being the predominant one. In cabbages, indolic compounds were the majority, with glucobrassicin and neoglucobrassicin being the major compounds [37].

All three types of samples analyzed, fresh useful, fresh residue, and blanched, presented the same profile of phenolic compounds. The useful parts and the leaves processed after cooking showed the highest concentrations of flavonoids, hydroxycinnamic acids, and total phenolic compounds, while the residues presented the lowest concentrations of both flavonoids and hydroxycinnamic acids. In turnip tops, 10 phenolic compounds were identified, with sinapic acid being the most abundant, followed by derivatives of kaempferol and isorhamnetin. In cabbages, 15 phenolic compounds were identified, including flavonoids and hydroxycinnamic acids.

In the cooking waters of cabbages and turnip tops, the highest content of total glucosinolates was found after approximately 1 h from the start of cooking. Aliphatic and indolic glucosinolates in the cooking waters presented different behavior, with aliphatic glucosinolates decreasing while indolic glucosinolates increasing their concentration.

The mentioned study by A Rosaleira company concluded that the residues of cabbages and turnip tops have a high content of glucosinolates, indicating that these waste products are interesting sources of these compounds to revalue their use and industrial utilization. On the other hand, the leaves of cabbages and turnip tops subjected to industrial blanching lose almost all the content of glucosinolates as these compounds degrade with heat. The loss of glucosinolates during blanching can be attributed to several reasons, such as the enzymatic action of myrosinase and high-temperature cooking, which can degrade the compounds. Additionally, the duration and specific blanching conditions can also influence the loss of glucosinolates. The residues of cabbages and turnip tops have lower concentrations of phenolic compounds than the fresh useful leaves. The fresh leaves of cabbages and turnip tops have a higher antioxidant capacity than the vegetable residues from the stems and older leaves although the residues still retain high concentrations, with approximately 25% loss compared to the useful leaves. The leaves of cabbages and turnip tops after industrial blanching preserve high values of antioxidant capacity, with minimal losses compared to the fresh useful material. The vegetable residues of cabbages and turnip tops, considered waste products during industrial packaging, constitute an important source of vitamins and minerals, with potassium content higher than even the leaves used for consumption. During blanching, the glucosinolates lost in the leaves of cabbages and turnip tops are gradually transferred to the cooking waters, reaching a maximum content after 45 min from the start of the industrial process. This would be the optimal time to collect the cooking waters for use in the agri-food industry [37].

Isothiocyanates and, particularly, sulforaphane have shown to influence epigenetically through various targets. A recent review on the role of sulforaphane as an anticancer agent emphasizes that this type of molecule can simultaneously modulate multiple cellular targets involved in carcinogenesis, including (1) modulating carcinogen-metabolizing enzymes and blocking the action of mutagens; (2) inhibition of cell proliferation and induction of apoptosis; and (3) inhibition of neo-angiogenesis and metastasis. Suforaphane targets cancer stem cells through modulation of nuclear factor kappa B (NF-κB), Sonic hedgehog (SHH), epithelial–mesenchymal transition, and Wnt/β-catenin pathways [97].

Therefore, some of the main epigenetic targets of sulforaphane are:

Histone deacetylases (HDACs): Sulforaphanes have been shown to affect histone modifications, such as acetylation and methylation, which can also influence gene activity. Sulforaphane has demonstrated inhibitory effects on histone deacetylases (HDACs), enzymes responsible for removing acetyl groups from histones [98,99]. By inhibiting these enzymes, sulforaphane can increase histone acetylation, which is associated with enhanced gene accessibility and increased gene expression.

DNA methyltransferases (DNMTs): Sulforaphane can also modulate the activity of DNMTs, enzymes responsible for DNA methylation. DNA methylation is a key epigenetic modification that can silence gene expression. It has been observed that sulforaphane can reduce DNMT activity, leading to a decrease in DNA methylation and potential reactivation of silenced genes [100]. Sulforaphanes may indirectly act by increasing the expression of demethylating enzymes, such as ten-eleven translocase (TET) [101], which are involved in the oxidation of methyl groups in DNA, potentially leading to reduced methylation in certain regions of the genome.

Sonic hedgehog (SHH): Dysregulation of the sonic hedgehog (Shh) signaling pathway has been associated with cancer stem cells (CSC) and implicated in the initiation of pancreatic [102], leukemia [103], and lung [104] cancers. Rodova and coworkers proposed that pancreatic cancer preventative effects of sulforaphane may result from inhibition of the Shh pathway. Thus, sulforaphane potentially represents an inexpensive, safe, and effective alternative for the management of pancreatic cancer [102].

Wnt/β-Catenin: Colorectal cancer is most frequently driven by hyperactive Wnt/β-catenin signaling. Sulforaphane has been shown to inhibit cell growth and blocks Wnt/β-catenin signaling of colorectal cancer cells [105].

MicroRNAs (miRNAs): It has been observed that sulforaphane can modulate the expression of certain miRNAs, which may have epigenetic effects by regulating the expression of specific genes [106].

These epigenetic targets of sulforaphane suggest its ability to influence gene expression and modulate key biological processes. Additionally, at the genetic level, sulforaphanes interact with transcription factors, which bind to DNA and regulate gene transcription. Sulforaphane can influence the activity of epigenetic transcription factors, such as nuclear factor erythroid 2-related factor 2 (Nrf2) [107,108], and hypoxia-inducible factor 1 (HIF-1) [109]. These transcription factors can modulate the expression of genes involved in epigenetic processes, such as antioxidant response and cellular adaptation to stress. Nuclear factor erythroid 2-related factor (Nrf2) is an important regulator of cellular antioxidant defenses. Sulforaphane can activate the Nrf2-ARE (antioxidant response element related to nuclear factor erythroid 2-related factor) signaling pathway, which is involved in antioxidant response and cellular protection against oxidative stress. By activating this pathway, sulforaphane promote the expression of a variety of genes encoding antioxidant and detoxifying enzymes, thereby enhancing the body’s ability to neutralize free radicals and other toxic compounds.

**Figure 9:**
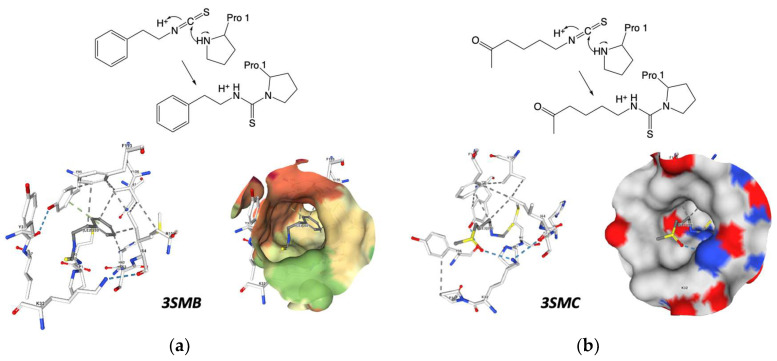
Crystallographic structures showing the interaction of: (**a**) 3SMB crystal structure: phenethylisothiocyanate covalently bound to macrophage migration inhibitory factor (MIF), pdb: https://doi.org/10.2210/pdb3SMB/pdb (accessed on 12 July 2023); (**b**) 3SMC crystal structure: macrophage migration inhibitory factor (MIF) with covalently bound L-sulforaphane. PDB: https://doi.org/10.2210/pdb3SMC/pdb (accessed on 12 July 2023) created with NGL [113].

Isothiocyanates also target macrophage migration inhibitory factor (MIF), a widely distributed protein known for its inflammatory, pro-tumorigenic, pro-angiogenic, and antiapoptotic properties. Isothiocyanates covalently inhibit MIF, providing insights into their cancer-preventive effects. Crichlow and coworkers present the crystallographic structures of human MIF bound to phenethylisothiocyanate and l-sulforaphane (Figure 9) [110].

**Figure 10:**
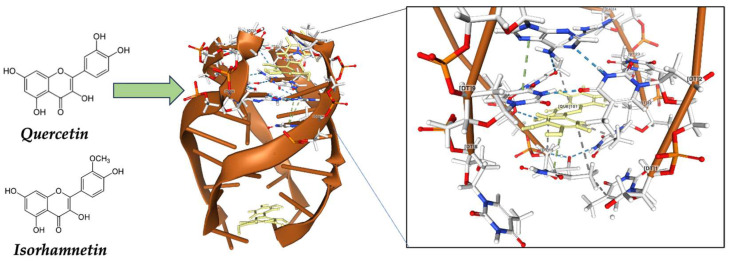
2MS6 crystal structure: human telomeric G-quadruplex DNA sequence (TTAGGGT)4 complexed with flavonoid quercetin. PDB: https://doi.org/10.2210/pdb2MS6/pdb (accessed on 12 July 2023) created with NGL [113].

Another predominant component in turnip tops is quercetin, a type of flavonoid (flavonol), with antioxidant activity found in a wide variety of plant-based foods (Figure 10). Quercetin has been shown to have antioxidant, anti-inflammatory, and antiproliferative properties [111,112], making it a bioactive compound with potential health benefits. Turnip tops are known to be a rich source of quercetin and other bioactive compounds. These compounds may contribute to the potential health benefits associated with turnip top consumption, such as protection against oxidative stress, a reduction in inflammation, and support for the immune system.

Regarding quercetin’s binding substrates, this molecule has been found to interact with various proteins and enzymes involved in key biological processes. Some known binding substrates of quercetin include

Protein kinases: Quercetin can inhibit the activity of certain protein kinases, which are enzymes involved in the regulation of cell growth and proliferation. This interference can affect cell signaling pathways and impact the growth of cancer cells.

ABC transporter proteins: Quercetin can interact with ATP-binding cassette (ABC) transporter proteins, which play a crucial role in drug resistance in cancer cells. By interfering with these proteins, quercetin may help overcome resistance to conventional treatments [114].

Inflammatory proteins: Quercetin has anti-inflammatory properties and can modulate the expression of inflammatory proteins, such as cytokines and transcription factors, which are involved in cancer progression [115].

Detoxification enzymes: Quercetin can induce the activity of detoxification enzymes, such as glutathione S-transferase (GST) enzymes, which play an important role in eliminating toxic compounds and carcinogens from the body [116].

Investigation into the utilization of anticancer agents that operate by enhancing the stability of telomeric G-quadruplex DNA has arisen as a fresh and captivating domain within the realm of discovering drugs for combating cancer. The G-quadruplex configuration plays a significant role in safeguarding the ends of chromosomes against undesired recombination and deterioration. Additionally, it hinders the operation of telomerase, the enzyme accountable for preserving telomere length. Protein Data Bank information about quercetin shows that targeting human telomeric G-quadruplex DNA could be one of the mechanisms by which this flavonoid exert anticancer activity. Figure 10 shows a co-crystal of quercetin with human telomeric G-quadruplex DNA sequence (TTAGGGT)4 and how this molecule can interact with DNA. It interacts with the telomeric sequence through π-stacking at two sites: between T1pT2 and between G6pT7 [117].

On the other hand, other important bioactive flavonoid found in turnip tops is isorhamnetin. It is a methylated derivative of quercetin. It has antioxidant and anti-inflammatory properties similar to quercetin.

It is important to note that the exact levels of quercetin analogous in turnip tops may vary depending on various factors, such as the type of plant, cultivation methods, and maturity of the sprouts. However, overall, “turnip tops” are considered a good source of quercetins and other beneficial nutrients for health.

**Scheme 4:**
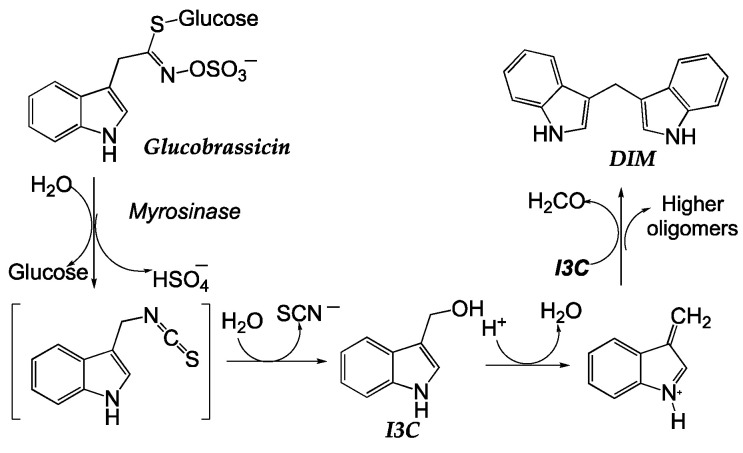
Hydrolysis of glucobrassicin glucosinolate, assisted by the enzyme myrosinase, yields I3C (indole-3-carbinol), followed by the subsequent biosynthesis of DIM (3,3’-diindolylmethane) [122].

In addition to sulforaphanes and flavonoids, diindolylmethane (DIM) and Indole-3-Carbinol (I3C) are two bioactive compounds found in brassicas such as broccoli, kale, cauliflower (widely consumed in the SEAD), and Brussels sprouts. These compounds are considered key components of brassicas due to their health-promoting properties and potential cancer-preventive effects [55,118,119]. I3C is a hydrolyzed glucosinolate product (Scheme 4). Cruciferous vegetables contain compounds such as sulforaphane and I3C [120]. When exposed to stomach acid pH, I3C is transformed into various DIM products [121].

The interest in phytonutrients extracted from cruciferous vegetables dates back twenty years when it was observed that broccoli, DIM, and I3C prevented chemically induced breast cancer in animals. DIM is formed from I3C through a condensation reaction in the gastrointestinal tract during the digestion of brassicas. DIM has been extensively studied for its antioxidant, anti-inflammatory, and anticancer properties. They modulate different biological pathways involved in cell growth, proliferation, apoptosis, and angiogenesis, which may help prevent tumor formation and growth. On the other hand, I3C has also been the subject of numerous studies due to its anticancer properties. I3C can modulate the expression of genes related to detoxification and elimination of carcinogens, as well as inhibit the growth of cancer cells [123]. Additionally, I3C has shown anti-inflammatory and antioxidant effects that can contribute to its anticancer activity. Both DIM and I3C have demonstrated beneficial effects in the prevention and treatment of different types of cancer, such as breast cancer, prostate cancer, and colon cancer.

**Scheme 5:**
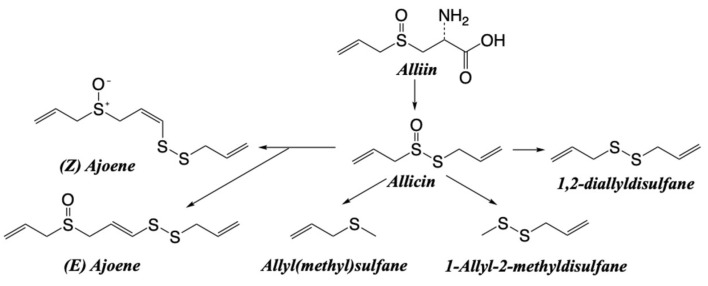
Biosynthetic pathway for the conversion of components in garlic from alliin.

Garlic (*Allium sativum*) belongs to the Lily family (Liliaceae). It is cultivated for the bulb that forms at the base of the leaves and on the stem, which has a disc-like shape. Garlic is a widely used food in the SEAD, especially in the Portuguese region, where frying is a dominant culinary technique. It has numerous health-beneficial properties and has been extensively investigated for its potential health benefits, including the anticancer effects of some of its bioactive components (Scheme 5) [124,125]. Garlic contains several bioactive compounds, including:

Ajoene: An organosulfur compound associated with anticancer properties. Ajoene has been shown to inhibit the growth of cancer cells in vitro and in animal models [126,127].

Allicin: Another organosulfur compound that is formed when garlic is cut or crushed. Allicin has antioxidant properties and has demonstrated antitumor effects in preclinical studies [128].

Diallyl sulfide: A garlic-derived compound with antiproliferative and proapoptotic properties. It has been studied for its potential in preventing and treating cancer [129].

When garlic is mechanically crushed or cut, the enzyme alliinase stored in the vacuoles of the garlic cells is released. This enzyme breaks the carbon-sulfur bonds of alliin, transforming it into the thiosulfonate allicin, which is unstable. The instability of allicin leads to its rapid degradation into hundreds of sulfonated compounds.

The potato (*Solanum tuberosum* L.) is the tuber of the plant with the same name, an herbaceous plant belonging to the Solanaceae family. It is very typical in the cuisine of the SEAD, being a key ingredient in many well-known dishes worldwide. In Galicia, it is commonly used in dishes such as “*cocido gallego*,” “*caldo gallego*,” “*carne o caldeiro*,” and “*caldeirada*” of fishes. In the northern region of Portugal, it can be found in dishes such as “*Bacalhau à brás*,” “*Cozido à portuguesa*,” or “*caldo verde*.” Potatoes are also featured in renowned dishes such as “*Fabada*” in Asturias or “*Botillo*” in El Bierzo.

**Figure 11:**
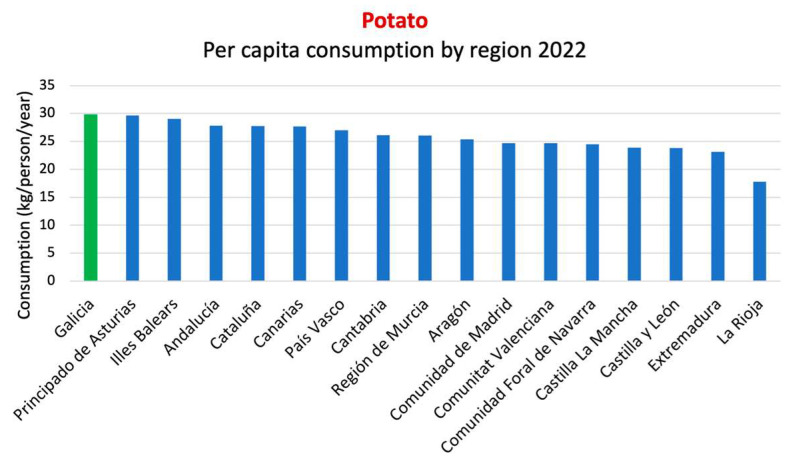
Own elaboration. Source: Ministry of Agriculture, Fisheries, and Food, Government of Spain, report on food consumption in Spain 2022 [14].

Galicia is the Spanish autonomous community with the highest per-capita consumption of potatoes, with individuals consuming 33.27 kg per year, compared to the national average of 28.89 kg (Figure 11). The cultivation of potatoes in Galicia is a Protected Geographical Indication (PGI), *“PGI Patatas de Galicia”* which bestows excellence on the potatoes produced in this region. Within this PGI, three main varieties are found: *Kennebec*, *Agria*, and *Fina de Carballo* [130].

Potatoes have been recognized by the Food and Agriculture Organization of the United Nations (FAO) as a staple and sustainable food for the growing global population [131]. Potatoes are rich in vitamin C, B-group vitamins, and heart-healthy minerals such as potassium. The primary culinary technique in the SEAD is boiling, which is considered the healthiest and main method of preparing potatoes, leaving the skin on to retain its bioactive molecules.

Studies have also explored the antioxidant properties of potatoes [132]. In addition to vitamin C, potatoes are a source of other antioxidant molecules such as carotenoids and phenylpropanoids, and they contain a variety of secondary metabolites. Carotenoids are isoprenoid-based molecules synthesized in plastids with a polyene backbone consisting of conjugated C=C bonds.

**Figure 12:**
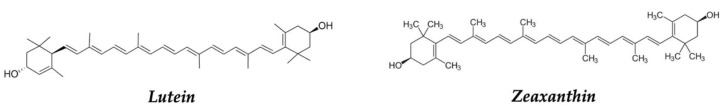
Chemical structures of lutein and zeaxanthin.

The carotenoids content differs in each potato variety, which is evident in their color. Zeaxanthin is the carotenoid responsible for the orange color, while lutein is responsible for the yellow color (Figure 12). The Y locus encodes a β-carotene hydroxylase, which is a key determinant of the pulp color of the tuber [133,134]. Potatoes exhibit a great diversity of carotenoids, many of which are strong singlet oxygen quenchers and eliminators of other reactive oxygen species (ROS) [135].

In the northern Portuguese region, where dishes are often accompanied by white rice rather than potatoes, the more reddish-colored potato is preferred, with the Desiree variety being predominant, a result of mixed varieties. In general, potato cultivation in Portugal is characterized by the reddish-colored potato, a variety called Red Lady.

**Galician Peppers** (*Capsicum annuum* L.): In the Galician region of the SEAD, five native varieties of peppers have been awarded different quality seals: “PGI Pemento da Arnoia,” “PGI Pemento do Couto,” “PGI Pemento de Oímbra,” “PGI Pemento de Mougán,” and the famous Herbón peppers (Pimientos de Padrón or Padrón pepper) with the “PGI Pemento de Herbón” quality seal. The importance and relevance of these varieties are reflected in several quality seals, including four Protected Geographical Indications (PGI) and one Protected of Origin Designation (POD) for the Padrón peppers. They each have their respective regulatory boards that ensure the preservation of the characteristics of Galician peppers [136].

**Figure 13:**
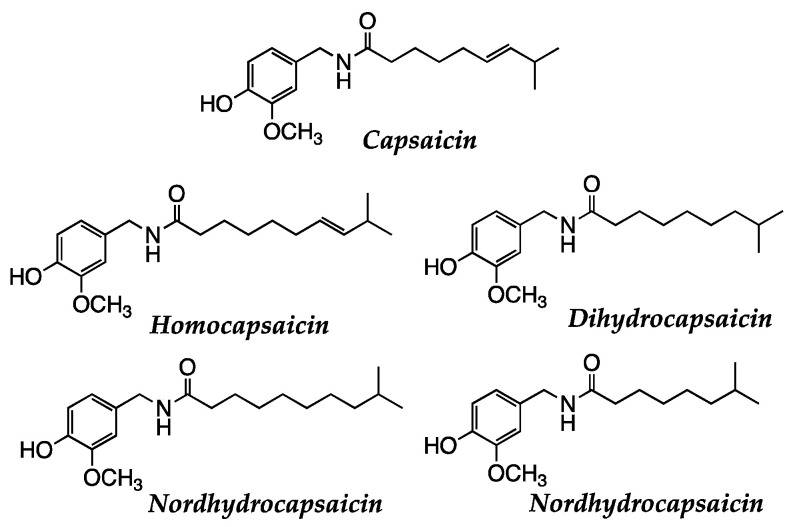
Chemical structure of capsaicin and other derivatives in *Capsicum annuum* L.

The Padrón pepper, of Mexican origin, arrived in Galicia through a pilgrimage carried out by Franciscan monks. It is known for its slightly spicy flavor, but in general, it is considered to have a mild taste. Capsaicin is a bioactive compound found in spicy peppers, including Padrón ones. It is responsible for their characteristic spicy flavor and has been studied for its potential beneficial health properties. Nevertheless, the capsaicin content is low compared to other spicier peppers. Capsaicin, dihydrocapsaicin, and some carotenoids are reported as the major active compounds with several pharmacological potentials, especially as anticancer and cardioprotectant [137] (Figure 13).

Padrón peppers also contain various antioxidants, such as carotenoids (such as b-carotene), vitamin C, and vitamin E. These antioxidants help protect cells from oxidative stress and contribute to overall health. It is essential to note that the levels of components may vary depending on the Padrón pepper variety, growing conditions, and stage of maturity. In this variety of peppers, we can find capsaicin (close to 50%), dihydrocapsaicin (between 30–40%), nordihydrocapsaicin (less than 10%), homodihydrocapsaicin and homocapsaicin (residuals) (Figure 13).

Capsaicin has been the subject of numerous scientific studies, and it has been shown to possess antioxidant, anti-inflammatory, and analgesic properties. Additionally, there have been suggestions that capsaicin may have anticancer properties due to its ability to inhibit the growth of cancer cells and stimulate apoptosis (programmed cell death) in certain types of cancer [138,139,140,141,142], as mentioned above. Several in vitro and animal studies have investigated the effect of capsaicin on different types of cancer, including prostate [143], colon [144], lung, and breast cancers, and others [145]. These studies have shown promising results, but further research and clinical studies in humans are needed to confirm these findings. It has also been suggested that capsaicin could have inhibitory effects on angiogenesis, the process by which tumors develop new blood vessels to support their growth.

A study by Clark highlights that capsaicin exhibits strong anticancer activity by targeting multiple signaling pathways and cancer-associated genes in different tumor stages, including initiation, promotion, progression, and metastasis. The anticancer mechanisms of capsaicin include the activation of apoptosis, cell growth arrest, and inhibition of angiogenesis and metastasis. Based on these results, the researchers concluded that capsaicin interacts synergistically with other cancer-preventive agents, providing the possibility for its potential use in cancer therapy along with other chemotherapeutic agents [146]. It is essential to note that the effects of capsaicin can vary depending on the dosage and the form of consumption. In the case of Padrón peppers, their content of capsaicin is generally low, which means that the potential health benefits associated with capsaicin may be limited in this specific case.

#### 2.3.3. Native Olive Oil

*Olea europaea* L. arrived in Galicia during the Romanization of the Iberian Peninsula in the 1st century, finding in “Gallaecia” (a Roman province that encompassed the territories of the current autonomous community of Galicia, northern Portugal, and the territories of the current provinces of León, Zamora, and the autonomous community of Asturias) an ideal climate for olive cultivation. The native olive oil from Galicia, known as Galician olive oil, is a product of high quality and uniqueness produced in the northwest region of Spain. Although Galicia is not one of the main olive oil-producing regions in Spain, it has native olive varieties that adapt to its specific climate and soil, giving rise to olive oil with distinctive characteristics. Despite its limited production, olive oil consumption in the Atlantic northwest within the SEAD is high, even higher than in the Mediterranean diet, being a characteristic food of this diet. Currently, in Galicia, its consumption is 16 g per person per day [147].

In Galicia, a recent study carried out by the Biological Mission of Galicia belonging to the Spanish National Research Council (CSIC) has identified 20 olive varieties, making it an olive-growing region with a humid climate and characteristic soil, resulting in a variety of olives subject to international attention [148].

Two of the most prominent varieties are “Brava Gallega” and “Mansa Gallega.” Galician olive oil is produced in small quantities and is renowned for its quality, freshness, and unique organoleptic characteristics. Its cultivation is done on terraces and hillsides, taking advantage of the region’s topography. Additionally, an early harvest of the olives is carried out to preserve their properties and obtain higher-quality olive oil. This extra virgin olive oil, considered a gourmet product, has a high content of monounsaturated fatty acids and polyphenols, which confer antioxidant and anti-inflammatory properties. These bioactive compounds are mainly found in the unsaponifiable part and play a preventive role in cardiovascular diseases and reducing oxidative stress, proliferation of tumor cells, and cell cycle progression. Its consumption is recommended both raw, as a salad dressing, and to enhance the flavor of cooked dishes, such as fish, seafood, or vegetables.

In the Portuguese part of the SEAD, we find an extra virgin olive oil with a quality seal, the “POD Trás-os-Montes.” In the region of Trás-os-Montes (Portugal), there is a high number of organic farmers, and the area also presents climatic, topographic, and edaphological differences that contribute to agricultural diversity. For cosmetic use, several studies have demonstrated potential antigenotoxicity in its ingredients.

**Figure 14:**
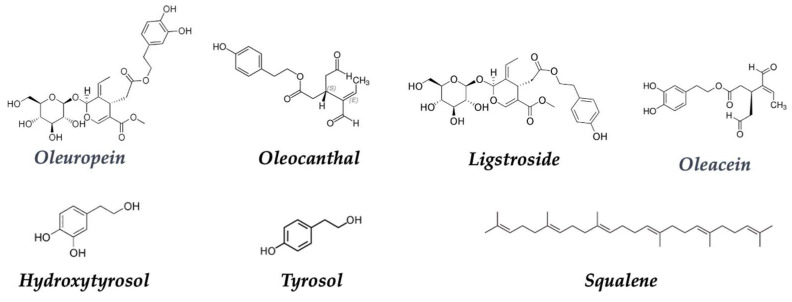
Some of the main active principles of olive oil.

Today, there is substantial evidence of the benefits of incorporating olive oil into the diet. Particularly, an excellent review on this topic has been published by Borzi and collaborators about olive oil effects on colorectal cancer. The interaction between gut microbiota and olive oil consumption could modulate colonic microbial composition or activity, with a possible role in cancer prevention [149]. Virgin olive oil (VOO) phenolic compounds, minor components of this fat, are known to be responsible for diverse health benefits when consumed in a regular diet. These benefits are mostly related to phenols such as tyrosol and hydroxytyrosol and secoiridoid derivatives such as ligstroside, oleuropein, oleocanthal, and oleacein, biomolecules found in extra virgin olive oil of the SEAD (Figure 14):

Hydroxytyrosol: A polyphenol present in extra virgin olive oil that has demonstrated antioxidant, anti-inflammatory, and anticancer properties [150,151,152]. It has been associated with the inhibition of cancer cell growth and induction of apoptosis (programmed cell death). A study showed that hydroxytyrosol has antiproliferative and proapoptotic properties in different tumor cells, and it suggests that extracellular production of hydrogen peroxide could be involved in these effects [153].

Oleocanthal: A phenolic compound found in extra virgin olive oil that has been associated with anti-inflammatory and anticancer properties. It has been shown that oleocanthal induces selective death of cancer cells without harming healthy cells. Experiments based on in vitro HT-29 human colon adenocarcinoma cells and in vivo chorioallantoic membrane assays showed an antitumor effect (due to increased AMPK) and apoptosis due to increased caspase-3 and poly-adenosine diphosphate-ribose polymerase, phosphorylation of p53 (Ser15)], and disruption of DNA [154].

Oleuropein: Another polyphenol present in extra virgin olive oil with antioxidant and anti-inflammatory properties. It has also been investigated for its anticancer potential due to its ability to inhibit the growth of tumor cells and stimulate apoptosis. In vitro studies using HT-29 human colon adenocarcinoma cells showed a decrease in cell proliferation after administration of this polyphenol to cells through activation of p53 pathway and #HIF-1 [155,156].

Ligstroside: A phenolic glucoside found in extra virgin olive oil. It has been shown that ligstroside has antioxidant properties and can help prevent the proliferation of cancer cells [157,158].

Squalene: A triterpene present in extra virgin olive oil that has demonstrated antioxidant and anticancer properties. Its potential for inhibiting the growth of cancer cells and preventing tumor formation has been investigated [159] confirming a chemopreventive behavior [160].

These molecules, along with other components of olive oil, work synergistically to provide health benefits and potential anticancer activity. It is essential to note that research in this field is still ongoing, and more studies are needed to fully understand the mechanisms of action and the effects of olive oil in cancer prevention and treatment [47,161,162].

#### 2.3.4. Native Chestnut of the SEAD

The predominant chestnut variety in Galicia is known as “*Castanea sativa*,” commonly referred to as Spanish chestnut or Galician chestnut. The chestnut is one of the foods that have a quality seal, the “PGI Castaña de Galicia” (Protected Geographical Indication of Galician Chestnut). The preservation of traditional chestnut cultivation techniques in Galicia has allowed for the selection of a homogeneous set of native cultivars, contributing to the current quality of Galician chestnuts [163].

Chestnut consumption is a deeply rooted tradition in Galicia, especially during the autumn season. Chestnuts are consumed in various forms, whether roasted, boiled, mashed, or in sweet preparations such as desserts.

Chestnuts are a good source of complex carbohydrates, dietary fiber, proteins, vitamins (mainly vitamin C and some B-complex vitamins), and minerals such as potassium, selenium, magnesium, and phosphorus. They are also low in fat and cholesterol-free.

**Figure 15:**
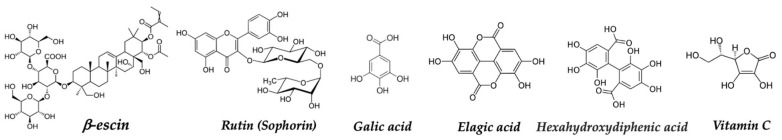
Main biomolecules with anticancer and antioxidant profile of chestnuts.

Chestnuts contain several bioactive components that have been of scientific interest (Figure 15). Many in vivo and in vitro studies have demonstrated how chestnut shell extract functions as an antioxidant and exhibits anticancer, anti-inflammatory, antidiabetic, and antiobesity activities [164].

Some of the main bioactive components of chestnuts are:

Tannins: Chestnuts contain specific tannins known as hydrolysable tannins, which are a type of polyphenol. These tannins are mainly found in the shell or outer skin of the chestnut. Hydrolysable tannins have a complex chemical structure, as they are formed by glucose units linked to ellagic acid or its derivatives. These units can be joined in the form of dimers, trimers, or larger polymers. The hydrolysable tannins in chestnuts have antioxidant properties, allowing them to neutralize free radicals and protect cells from oxidative damage. Additionally, they have demonstrated potential as anticancer agents [165]. It is important to note that hydrolysable tannins in chestnuts are present in higher quantities in the outer shell, so direct consumption of chestnuts, such as roasted chestnuts, does not entail a significant intake of these compounds. Hydrolyzable tannins present in chestnuts are mainly composed of ellagic acid and its derivatives, such as gallic acid and hexahydroxydiphenic acid (HHDP). These tannins are responsible for the astringent and antioxidant properties of chestnuts.

Escin: It is a compound found in the seeds of the horse chestnut tree (*Aesculus hippocastanum*). It has been traditionally used in herbal medicine, and its properties and potential applications have been studied [166,167,168]. Escin has demonstrated anti-inflammatory and venotonic properties, that is, it can help strengthen blood vessels and reduce inflammation. Due to these properties, escin has been used in the treatment of venous disorders, such as varicose veins and chronic venous insufficiency. Escin is a group of steroidal saponins found in various plant species. Its potential anticancer properties have been investigated due to its anti-inflammatory, antioxidant, and antiproliferative properties [166,167]. Escin has also shown inhibitory effects on the growth of cancer cells in laboratory studies and animal models. It is believed to exert its anticancer action through different mechanisms, such as inducing apoptosis (programmed cell death), inhibiting angiogenesis (formation of new blood vessels in tumors), and suppressing inflammation [168].

Ellagic Acid: This is another phenolic compound present in chestnuts with antioxidant and anticancer properties [169]. Ellagic acid has been shown to have potential anticancer effects by inhibiting the growth of cancer cells and promoting apoptosis (programmed cell death) [170].

In addition to ellagic acid and hydrolyzable tannins, chestnuts contain other phenolic compounds and phytochemicals. Some of these compounds are:

Gallic Acid: It is a phenolic acid present in chestnuts and many other plants. It has antioxidant properties and has been associated with potential health benefits [171].

Quercetin: It is a flavonoid found in some varieties of chestnuts. As discussed earlier, quercetin is known by its antioxidant and anti-inflammatory activity, and it has been associated with various beneficial effects on health.

Rutin: Another flavonoid present in chestnuts. Rutin also possesses antioxidant properties and has been studied for its protective effects on blood vessels and its potential to improve circulation [172].

Vitamin C: Chestnuts also contain vitamin C, although in smaller amounts compared to other fruits. Vitamin C is an essential antioxidant that contributes to immune function and the maintenance of healthy tissues.

#### 2.3.5. Autochthonous Honey in the SEAD

**Figure 16:**
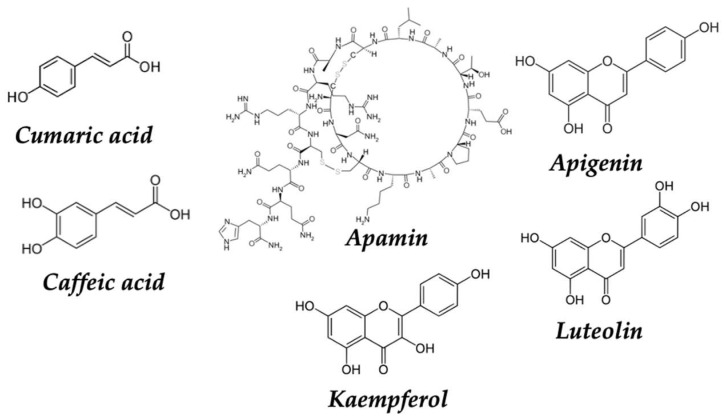
Most significant biomolecules found in honey.

The predominant autochthonous honey in the SEAD is known as “PGI Miel de Galicia” with its quality food seal. The peculiarities of Galicia’s climate, soil, and vegetation give rise to diverse honeys with very different characteristics and flavors [173]. It contains a variety of bioactive components, at least 180, although these vary depending on many factors. Some of these components have been studied for their potential anticancer properties (Figure 16). Honey contains several phenolic compounds, such as phenolic acid, flavonoids, and caffeic acid. These compounds have antioxidant and anti-inflammatory properties, which are believed to play a role in protection against cancer. Honey also contains bioactive peptides, some of which have shown anticancer activity, such as apamin [174]. Below are some of the most important biomolecules in honey with potential anticancer properties, some of which have already been co-crystallized with some of their anticancer targets:

Apamin: Although apamin is technically a neurotoxin produced by bees, it has been shown to have antitumor properties in in vitro studies and animal models [175]. However, more research is needed to fully understand its mechanism of action and its potential clinical application.

Among the flavonoids present in honey with anticancer activity, the following can be mentioned:

Quercetin: It is one of the most studied flavonoids in honey and has been associated with anticancer properties, as mentioned earlier.

Luteolin: Luteolin is a flavonoid identified in certain honeys. It is a polyphenolic compound belonging to the class of flavones. The amount and presence of luteolin in honey can vary depending on the botanical source of the honey, as different types of flowers produce honeys with distinct chemical profiles [176,177]. Recent data demonstrate that luteolin induces apoptotic cell death via antioxidant activity, acting as an anticancer agent against various types of human malignancies, including breast cancer [178]. Therefore, luteolin can be considered a flavonoid with a multifaceted anticancer potential [179].

**Figure 17:**
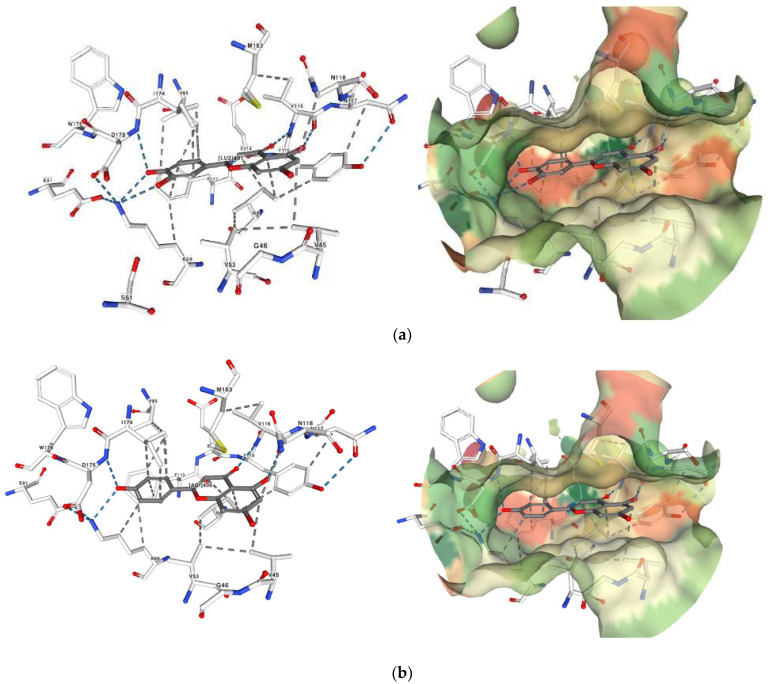
(**a**) 4DGN crystal structure: crystal structure of maize CK2 in complex with the inhibitor luteolinin, PDB: https://doi.org/10.2210/pdb4DGN/pdb (accessed on 12 July 2023). (**b**) 4DGM crystal structure: crystal structure of maize CK2 in complex with the inhibitor apigenin. PDB: https://doi.org/10.2210/pdb4DGM/pdb (accessed on 12 July 2023), created with NGL [113].

Apigenin: Another flavonoid found in honey and other plant sources is apigenin. It has been shown to possess anticancer activity by inhibiting angiogenesis (the formation of new blood vessels in tumors) and modulating different cell signaling pathways involved in the growth and proliferation of cancer cells, particularly protein kinase CK2 (‘casein kinase II’). The crystal structure of luteolin (Figure 17a) and apigenin (Figure 17b) in complex with the catalytic subunit of Zea mays CK2 has been solved. As seen, apigenin interacts with both the hinge region (Val116) and the positive area near Lys68 and the conserved water W1, the two main polar ligand anchoring points in the CK2 active site [180].

**Figure 18:**
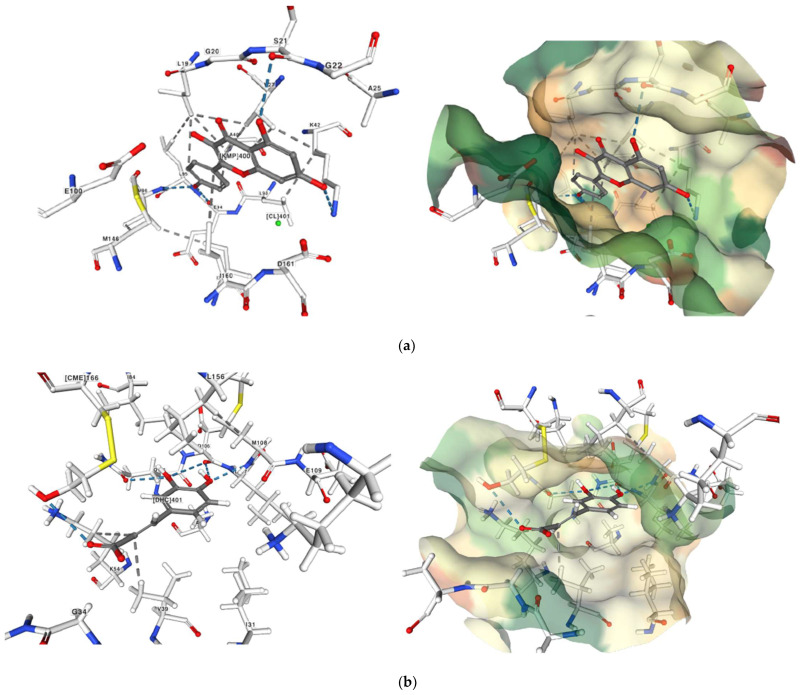
(**a**) 5AUX crystal structure: crystal structure of DAPK1 in complex with kaempferol, PDB: https://doi.org/10.2210/pdb5AUX/pdb (accessed on 12 July 2023). (**b**) 4N0S crystal structure: crystal structure showing the complex of ERK2 with caffeic acid. PDB: https://doi.org/10.2210/pdb4N0S/pdb (accessed on 12 July 2023), created with NGL [113].

Kaempferol: Kaempferol-rich foods have been related to a decreased risk of developing certain types of cancers, including skin, liver, and colon cancers. Kaempferol is a dietary anticancer molecule with multiple mechanisms of action [181]. Some of these include apoptosis, cell cycle arrest at the G2/M phase, downregulation of epithelial–mesenchymal transition (EMT)-related markers, and inhibition of phosphoinositide 3-kinase/protein kinase B signaling pathways [182]. Wang reported on the mechanism of anticancer action and the potential clinical use of kaempferol in the treatment of breast cancer [183]. It has also been observed that kaempferol induces apoptosis in cancer cells and can inhibit their proliferation and migration. Death-associated protein kinase 1 (DAPK1) is a 160 kDa serine/threonine protein kinase that belongs to the Ca(2+)/calmodulin-dependent protein kinase subfamily [184] (Figure 18a).

Phenolic Acids: Honey contains several phenolic acids, such as caffeic acid and p-coumaric acid, which have also been investigated for their potential anticancer activity. These compounds have shown inhibitory effects on the growth of cancer cells and may possess antioxidant and anti-inflammatory properties. For example, caffeic acid significantly inhibits colony formation of human skin cancer cells. Topical application of caffeic acid to dorsal mouse skin significantly suppresses tumor incidence and volume in a solar UV (SUV)-induced skin carcinogenesis mouse model. Caffeic acid directly targets protein-serine/threonine kinase ERK1/2 inhibiting them in vitro. The cocrystal structure of ERK2 complexed with caffeic acid is shown in Figure 18b. These results suggest that caffeic acid exerts chemopreventive activity against SUV-induced skin carcinogenesis [185].

#### 2.3.6. Wines

Both Galicia and Northern Portugal regions boast a long winemaking tradition, a wide variety of native grape varieties, and a climate and soils conducive to producing quality wines. This is why Galicia has five prestigious POD, such as “POD Rías Baixas”, “POD Ribeiro”, “POD Valdeorras”, “POD Ribeira Sacra”, and “POD Monterrey”, which have gained international recognition and great prestige [90]. In Northern Portugal, the most prominent PODs are Vinho Verde, Douro, and Dão. Vinho Verde is well recognized, while Douro is renowned for its production of Porto wines, as well as red wines.

Within the framework of the SEAD, each designation of origin in Galicia has unique characteristics due to its Atlantic climate, ocean influence, and specific soils in each area. Certain specific substances contribute to the mentioned profiles in Galician wines. These present a wide range of aromatic compounds, such as esters, aldehydes, terpenes, and phenolic compounds. These compounds come from the grape varieties used, such as Albariño, Treixadura, Caiño, Godello, Loureiro, or Mencía [186], and they provide specific organoleptic characteristics to these wines. The acidity in Galician wines is primarily attributed to the presence of tartaric and malic acids.

**Figure 19:**
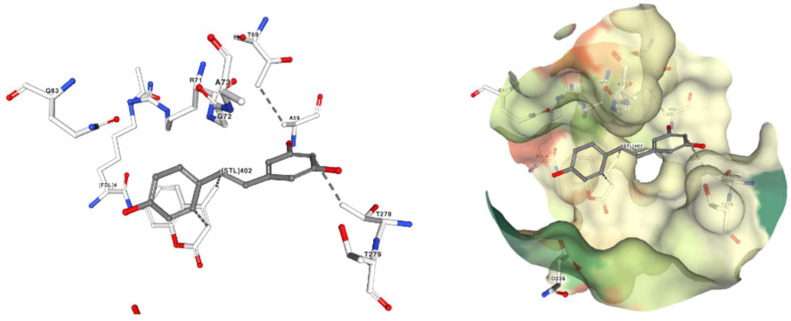
4HDA crystal structure: crystal structure of human Sirt5 in complex with Fluor-de-Lys peptide and resveratrol. PDB: https://doi.org/10.2210/pdb4HDA/pdb (accessed on 12 July 2023), created with NGL [113].

The granite and slate soils present in Galicia can influence the mineral profile of the wines. While there are no specific mineral substances directly responsible for this character, it is believed that the interaction of vine roots with these soils can influence mineral absorption and, ultimately, the expression of terroir in the wines. As a result, Galician wine contains a variety of bioactive components, including resveratrol (Figure 19), which is widely recognized for its beneficial health properties.

**Figure 20:**
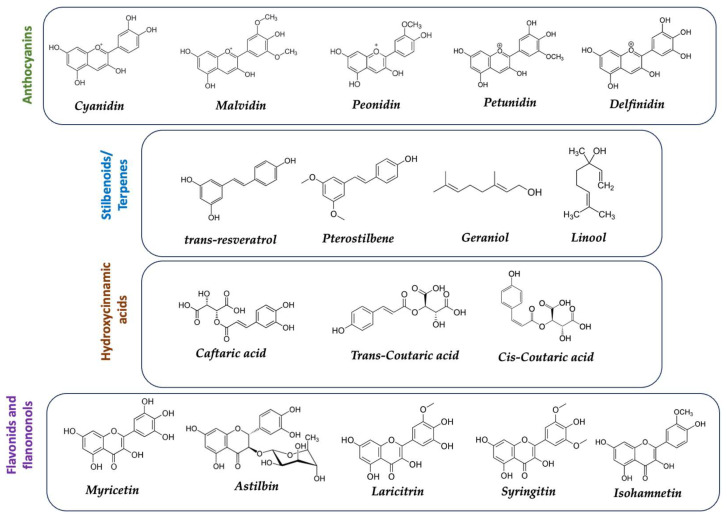
Some of the main bioactive components of Galician wines.

Native grape varieties, such as Albariño, Godello, and Mencía, have unique chemical compositions. For example, Albariño is characterized by a high content of aromatic compounds, such as geraniol and linalool (Figure 20), which contribute to distinctive floral notes. Godello is known for its good acidity and aromatic profile of white and citrus fruits. Mencía, used in red wines, presents phenolic compounds that provide color and structure to the wines.

Resveratrol is a polyphenol found in grape skins and has been associated with antioxidant, anti-inflammatory, and cardiovascular properties. The Mencía grape variety, one of the most cultivated red grapes in Galicia, is known to be particularly rich in resveratrol. Moderate consumption of wine rich in resveratrol has been suggested to contribute to reducing the risk of cardiovascular diseases and have positive effects on health, including anticancer therapy [187,188]. Particularly, resveratrol has been the subject of numerous studies due to its potential anticancer activity [189]. Therefore, polyphenols of Galician wines have antioxidant properties and may contribute to cancer prevention through moderate wine consumption [190]. Some of the pharmacological targets identified for resveratrol include

AMP-activated protein kinase (AMPK): Resveratrol can activate AMPK, an enzyme that regulates cellular metabolism and survival. AMPK activation can inhibit the growth of cancer cells and promote apoptosis (programmed cell death) [187].

**Figure 21:**
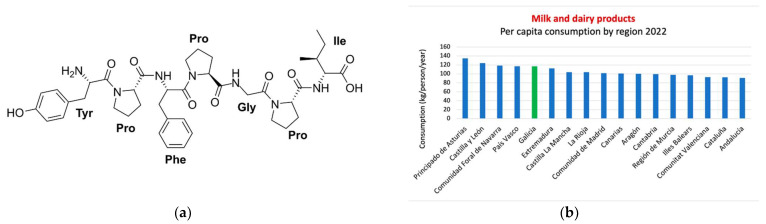
(**a**) Chemical structure of β-casomorphin. (**b**) Milk and dairy derivatives consumption in Spain and Galicia (green bar). Self-made. Source: Ministry of Agriculture, Fisheries and Food, Food consumption report of Spain, 2022, Government of Spain. Source: [14].

Sirtuin family proteins: Resveratrol can activate sirtuins. In fact, a crystallographic structure, available at PDB, shows the Sirt5-*trans*-resveratrol interaction (Figure 21). Sirtuins are a class of enzymes involved in the regulation of aging and cellular metabolism [191]. Sirtuins can influence the expression of genes related to cancer and modulate different cellular processes. Sirtuin enzymes have been studied since then. This family of seven proteins known as the “longevity gene” plays an important role in the aging process, protecting cells and performing other functions in our body. Sirtuins protect against oxidative stress, repair our DNA making it more resistant to genetic toxicity, and activate metabolism to burn fat, i.e., they combat metabolic-energetic stress.

Nuclear factor kappa B (NF-κB): Resveratrol can inhibit the activation of NF-κB, a transcription factor that plays an important role in the regulation of the inflammatory response and cell survival [192]. Inhibition of NF-κB can reduce proliferation and promote apoptosis in cancer cells.

Estrogen receptor (ER): Resveratrol can interact with the estrogen receptor, which is involved in the growth and proliferation of certain hormone-sensitive cancers, such as breast cancer. Resveratrol can have estrogenic or antiestrogenic effects depending on the context and concentration [193].

In addition to resveratrol, Galician wine contains other polyphenols (Figure 20), such as flavonoids (such as catechins, proanthocyanidins, or quercetin), phenolic acids (ellagic acid), and stilbenes (such as caffeic and gallic acid), some of which have been mentioned previously.

In this regard, several studies have analyzed Galician wines. Figueiredo-González reported the evolution of anthocyanins and flavonols in red grapes of *V. vinifera* L. *Mouratón*, collected separately from the tip and shoulder positions of the bunch, and during over ripening [194]. Derivatives of five anthocyanins (malvidin, peonidin, petunidin, delphinidin, and cyanidin) and derivatives of six flavonols (quercetin, myricetin, kaempferol, laricitrin, isorhamnetin, and syringetin) were detected (Figure 20).

Another recent study by Sonia Sentellas and coworkers analyzed the combined extraction and purification process and demonstrated that malolactic fermentation lees of Albariño wines (white wine) are a valuable source of phenolic compounds, especially phenolic acids such as caftaric acid, cis and trans-coutaric acid (Figure 20) [195].

Mirás-Avalos reported the significance of amino acids in wine quality and their potential for classifying wines based on their variety. In this research, the amino acid compositions of Albariño, Godello, and Treixadura wines were analyzed. The study found that the most abundant amino acids present in these wines were proline, aspartic acid, glutamic acid, lysine, arginine, asparagine, alanine and histidine [196].

The grape seeds, along with the grape skins, are one of the parts that are discarded during the wine-making process, posing a challenge for many wineries. Scientific studies have shown that grape seeds contain bioactive compounds that could be beneficial for human health [197]. With the objective of utilizing these residues, scientists from the CSIC at the Biological Mission of Pontevedra have extracted oils from the grape seeds that remained as waste after the production of white and red wines from Galician grape varieties (vitis vinifera) such as Albariño, Caíño Blanco, Loureiro, and Mencía [197].

It has been found that each of these oils, depending on the grape variety they come from, has different concentrations of vitamin E, omega-3 fatty acids, and phenolic compounds called proanthocyanidins, with potential anticancer and antimetastatic effects [198], as well as favorable effects in diseases such as diabetes.

Grape seed oil provides vitamin E and has a high concentration of linoleic acid (76%) and linolenic acid, essential fatty acids also known as omega-6 and omega-3, which are crucial for the synthesis of prostaglandins, substances necessary to reduce platelet aggregation and inflammation. It serves as an ally in maintaining cardiovascular health and helps in the prevention of hypertension, obesity, and diabetes [199].

#### 2.3.7. Milk and Dairy Derivatives

The consumption of milk and its derivatives, especially cheese, is higher than that of the Mediterranean diet, with Galicia being one of the regions in Spain, where per-capita consumption is particularly elevated (Figure 21). Milk peptides are short sequences of amino acids naturally found in milk, which may have bioactive effects in the body. Some of these peptides have shown antitumor activity or are associated with anticancer properties:

Lactoferricin: Derived from lactoferrin, a protein present in milk, lactoferricin has demonstrated antitumor activity by inhibiting the growth of cancer cells and promoting apoptosis. It has also been observed to modulate the immune response and possess anti-inflammatory properties [200].

Casomorphins: These are opioid peptides found in casein, a protein present in milk. Some studies have suggested that casomorphins may have antiproliferative effects on cancer cells, especially in colon cancer [201]. However, further research is needed to fully understand their anticancer activity. The interaction of opioid peptides with cancer cells has been reported to exhibit anticancerous activity. Specifically, T47D human breast-cancer cells cultured with or without in culture express opioid receptors and opioid agonists. The effect of suppression of T47D cell proliferation by opioids is achieved via k- and d-opioid receptors. The mechanisms through which these opioid-like molecules stimulate the anticancer effect are probably via disassembling the actin microfilaments with the activation of focal adhesion kinase (FAK) phosphorylation and vinculin with substantial small GTPase Rac1 activation [202].

Alpha-lactalbumin: This peptide is present in the lactalbumin protein. Alpha-lactalbumin has been shown to possess antitumor properties by inhibiting the growth of cancer cells and promoting apoptosis [203]. It has also been suggested to modulate the immune response and exhibit anti-inflammatory properties [204].

#### 2.3.8. Dietary Supplementation in the SEAD

The SEAD contains several bioactive molecules that provide great health benefits. Among them, we find hydroxytyrosol and oleic acid present in olive oil, a widely consumed food in this diet. A systematic review and meta-analysis evaluated the beneficial effects of dietary supplementation with olive oil, oleic acid, or hydroxytyrosol in metabolic syndrome [205]. There is also scientific evidence confirming the health benefits of supplementing the Mediterranean diet with extra virgin olive oil (EVOO). In a study with individuals at high cardiovascular risk, the incidence of major cardiovascular events was lower among those assigned to a diet supplemented with extra virgin olive oil or nuts compared to those on a low-fat diet [206]. In addition, a small crossover trial (Trial number: ISRCTN09220811) was conducted in the context of the “EUROLIVE” study with 200 participants who, after a two-week washout, consumed 25 mL of olive oil with different phenolic content three times a day for three weeks. The results indicated a linear decrease in the ratio of oxidative stress markers and total cholesterol/HDL-cholesterol matching the polyphenol content in olive oil [207].

The most characteristic condiment of the SEAD is red paprika, also known as paprika, which provides organoleptic and nutritional characteristics. It is present in many traditional dishes such as “pulpo a la gallega”, “carne o caldeiro”, or “caldeirada de pescado”. It is obtained from grinding dried red peppers, usually from the *Capsicum annuum* variety [208].

Red paprika contains various components that can provide health benefits due to their antioxidant properties, helping to neutralize free radicals and protect cells against oxidative damage, which may contribute to the development of diseases, including cancer. Some of these bioactive molecules include:

Capsaicinoids: These compounds are responsible for the spicy flavor of red paprika. Capsaicinoids have been shown to possess antioxidant, anti-inflammatory, and analgesic properties as already mentioned, and they can help improve cardiovascular health [209].

Carotenoids: Red peppers, from which red paprika is derived, are rich in carotenoids such as beta-carotene and capsanthin. These compounds act as antioxidants and are converted into vitamin A in the body, contributing to skin health, vision, and immune system function.

Vitamin C: Red paprika is a good source of vitamin C, an essential antioxidant that contributes to immune function, collagen formation, and protection against oxidative damage, as mentioned earlier.

In addition to paprika, some dishes in the SEAD are also supplemented with lard, which is consumed at higher rates in Galicia compared to the rest of Spain, giving a characteristic flavor to some of its dishes [210]. In northern Portugal, cinnamon or other herbs typical of the Mediterranean diet, such as cilantro, are also used.

Furthermore, the term “supplements” also encompasses dietary foods [211]. Dietary foods are those that have been modified in their original composition by adding, removing, or substituting some of their nutrients, such as carbohydrates, proteins, lipids, vitamins, and inorganic nutrients or minerals, and they are part of the usual diet.

Among the dietary foods modified by the food industry, many are tailored to address specific nutritional needs and requirements in certain conditions, such as gluten-free or low-gluten foods for celiac disease or gluten intolerance, lactose-free milk for lactose intolerance, and foods without added sugars for diabetes, or enriched with nutrients such as omega-3, vitamin D, calcium, or fiber.

There are also dietary supplements, which are products designed to complement the diet and provide additional nutrients. The population perceives them as safe, leading to increased self-prescription. These products come in various forms, such as tablets, capsules, powders, liquids, or bars, and they contain a variety of vitamins, minerals, herbs, amino acids, or other ingredients [212]. Different types of dietary supplements exist, with the most common being vitamins and minerals, which represent 50% of the supplements consumed in Europe [213]. They contain one or several essential vitamins and minerals, such as vitamin C, vitamin D, calcium, and iron. They are used to address specific nutritional deficiencies or to support certain life stages, such as pregnancy or lactation. It is advisable to be under medical supervision and to avoid self-prescription.

The consumption of probiotics has also increased. These are dietary supplements containing beneficial bacteria for the intestine. They help maintain a healthy balance of bacteria in the digestive tract and have been shown to have positive effects on intestinal health and immune function [121].

Regarding omega-3 fatty acids, it is important to remember that the SEAD is rich in fish and seafood, which are a source of this bioactive molecule.

Lastly, there are already antioxidant supplements on the market containing various bioactive molecules commonly found in the SEAD. For example, sulforaphane, found abundantly in vegetables of the *Brassica oleracea* genus, is marketed in doses of 20 mg, equivalent to almost three servings of broccoli (500 g). Hydroxytyrosol, highly present in extra virgin olive oil, is widely used in nutritional supplements in amounts ranging from 0.7 to 1.5 mg, prescribed for specific eye conditions such as macular degeneration or cataracts [214]. There are also supplements with a significant phytonutrient from brassicas, I3C, which acts as an anticancer molecule [215,216]. The most effective dose is 400 mg daily [217]. Finally, we find phenolic compounds extracted from seaweed that act as enzyme inhibitors and exhibit antimicrobial, antiviral, anticancer, antidiabetic, antioxidant, and anti-inflammatory activities [218]. Table 1 presents a summary of the foods and active compounds of the SEAD and their relationship with anticancer or antioxidant properties analyzed in this study and a summary of all discussed contents in this section can be seen in Table 2.

## 3. Conclusions

Current dietary patterns, especially among young people in European countries, are undergoing significant changes. The influence of globalization, urbanization, modern lifestyles, and the availability of processed foods has led to an increase in the consumption of foods high in saturated fats, added sugars, and ultra-processed foods, and a decrease in the consumption of fresh and healthy foods. This trend towards less healthy eating has concerning consequences for the health of young people and the population in general. The rise in obesity, cardiovascular diseases, diabetes, and other diet-related health issues is evidence of the need to take action to address this situation. In this context, there is a growing interest in returning to the Atlantic or Mediterranean dietary patterns, which have been associated with a range of health benefits.

These diets are characterized by high consumption of fruits, vegetables, legumes, whole grains, fish, dairy and dairy products, olive oil, and nuts, and moderate consumption of red meats and alcohol. The Atlantic diet has been extensively studied and has been shown to have positive effects in preventing chronic diseases, including cardiovascular diseases, type 2 diabetes, and some types of cancer. These eating patterns are rich in essential nutrients, antioxidants, healthy fatty acids, and bioactive compounds such as polyphenols and omega-3, which may have anti-inflammatory, antioxidant, and health-protective properties.

The following approaches could be considered in new research about the SEAD: investigate and characterize more natural antioxidants present in SEAD foods, such as polyphenols in wines and olive oil, and their ability to prevent oxidative stress and cell damage (1); develop more studies of compounds with anti-inflammatory properties in foods such as fish, seafood, and green leafy vegetables, and their impact on reducing chronic inflammation, a risk factor in the development of certain types of cancer (2); investigate how SEAD components can influence gene expression through epigenetics, especially in the regulation of genes related to cancer—this could include the identification of specific miRNAs and their response to SEAD foods (3); discover more specific molecules in SEAD foods that may have direct or indirect properties in inhibiting tumor growth or inducing apoptosis in cancer cells (4); investigate synergies between different components of the SEAD, such as the combination of certain foods or compounds that may have an even greater protective effect against cancer when consumed together than separately (5); conduct research to evaluate how SEAD components are metabolized by the body and how these metabolites may impact cancer prevention (6); conduct clinical trials that consider genetic variability and individual patient characteristics to assess the effectiveness of the SEAD in cancer prevention or adjunctive treatment (7); finally, conduct long-term studies that follow individuals adhering to the SEAD to assess cancer incidence over their lifetime and determine if there are sustained protective effects (8).

The review presented here gathers the established scientific evidence that demonstrates the connection between a healthy diet, exemplified by the SEAD, and the prevention of different types of cancer. The crystallographic structures available in PDB that evidence the component–target interaction are still somewhat scarce, which was a limitation in this study. Reverting to Atlantic dietary patterns involves promoting the consumption of fresh, minimally processed, and locally sourced foods while reducing the intake of processed and ultra-processed foods. This requires education and awareness about the health benefits of these eating patterns, as well as changes in the availability and accessibility of healthy foods.

## Figures and Tables

**Table 1:** Relationship between the main foods of the SEAD, their key components, and the reported pharmacological activity.

202Dietary Source	Main Bioactive Component	Activity	Reference
**Fish, shellfish, seaweed**	Fucoxanthin	Antioxidant	[24]
	Omega-3 fatty acids	Antioxidant, anti-inflammatory, hypocholesterolemic	[25]
	Selenium	Antioxidant	[59,60]
	Betaine (TMG, Anserine, Carnosine)	Anticancer, antioxidant, anti-inflammatory	[67,68]
	Vitamin D	Epigenetic modifications	[89]
**Octopus**	Selenium	Antioxidant	[59]
	Taurin	Antiproliferation	[74]
	OCTPEP-1	Anticancer	[83]
	Ozopromide	Antioxidant, anti-inflammatory	[87,88]
**Brassicaceae family**	Glucosinolate	Antioxidant, anti-inflammatory, anticancer	[37,92]
	Sulphorafane	Antioxidant, anti-inflammatory, anticancer	[37,96,97,98,99]
	Indole-3-carbinol	Anticancer	[55,117,123]
	Quercetin	Antioxidant, anti-inflammatory, anticancer	[111,112,115,117]
	3,3-Diindolylmethane	Anticancer	[120,121]
**Garlic**	Ajoene	Anticancer	[126]
	Allicin	Antioxidant, anticancer	[127]
	Diallyl sulfide	Antiproliferative, anticancer	[128,129]
**Potatoes**	Carotenoids	Antioxidant	[131,132,133]
	Vitamin C	Antioxidant	[131,132,133]
**Peppers of Padrón**	b-Caroten	Antioxidant, cardioprotective	[134]
	Capsaicine	Anticancer, anti-inflammatory	[138,139,140,141,142,143,144,145]
**Olive oil**	Hydroxytyrosol	Anticancer	[149,150,151]
	Oleocanthal	Anti-inflammatory	[154]
	Oleuropein	Anticancer	[155,156]
	Ligstroside	Antioxidant	[157,158]
	Squalene	Antioxidant	[158]
**Chestnuts**	Tannins	Antioxidant, anticancer	[164,165]
	Escin	Anticancer	[165,166,167]
	Ellagic acid	Antioxidant, anticancer	[169]
	Rutin	Antioxidant	[171]
**Honey**	Apamin	Anticancer	[173,174]
	Apigenin	Anticancer	[179]
	Kaempferol	Anticancer	[180,181,182,183,184]
**Wine**	Resveratrol	Anticancer	[187,188,189,190,191,192]
	Tannins	Antioxidant, anticancer	[164,165]
**Milk and dairy derivates**	Bioactive peptides	Antioxidant, anticancer	[200]
	Lactoferricin	Antioxidant, anticancer	[200]
	Casomorphins	Antioxidant, anticancer	[201]
	Alpha-lactalbumin	Antioxidant, anticancer	[203]
**Paprika, red pepper**	Zeaxanthin, lutein	Antioxidant	[132,133,207,208,209]
**Supplements**	Sulphorafane	Antioxidant, anti-inflammatory anticancer	[37,96,97,98,99]
	Zeaxanthin, lutein	Antioxidant	[132,133]
	Caspsaicinoids	Antioxidant, anti-inflammatory, anticancer	[208]
	Hidroxytyrosol	Antioxidant, anticancer	[213]
	Indole-3-carbinol	Anticancer	[214,215]
	Phlorotannins	Antioxidant, anticancer	[217]

**Table 2:** Summary of the discussed contents.

Dietary Source	Summary of Main Concepts
**2.3.1 Mostly consumed fish and a food in the SEAD: mussels, octopus and cod.**	→ Ozopromide showed proapoptotic effects on A549 lung cancer cells and inhibits pro-inflammatory markers. → Betaines can participate in DNA methylation and other molecules, which can regulate gene expression and other cellular processes. Anticancer activity of selenium is still controversial
**2.3.2 Genuine vegetables of the SEAD: Galician turnip top, garlic, potato, and Padrón pepper**	→ Glucosinolates are natural compounds found in plants of the Brassicaceae family and break down into biologically active compounds, such as sulforaphanes, when cut, chewed, or processed. Sulphorafane targets histone deacetylases (HDACs, DNA methyltransferases (DNMTs), Sonic hedgehog (SHH): Wnt/β-Catenin: microRNAs (miRNAs). Sulforaphane can influence the activity of epigenetic transcription factors, such as nuclear factor erythroid 2-related factor 2 (Nrf2). → Indole-3-carbinol and 3,3-diindolylmethane (DIM) modulate different biological pathways involved in cell growth, proliferation, apoptosis, and angiogenesis. DIM suppressed mRNA and protein expression of DNM1, DNM3a, and DNMt3b; inhibited protein expression of HDAC2 and HDAC3. → Quercetin can induce the activity of detoxification enzymes, such as glutathione S-transferase (GST) enzymes. Quercetin also targets human telomeric G-quadruplex DNA sequence (TTAGGGT)4. → Capsaicine exhibits strong anticancer activity by targeting signaling pathways and cancer-associated genes in different tumor stages, including initiation, promotion, progression, and metastasis. Allicin has antioxidant properties and has demonstrated antitumor effects in preclinical studies.
**2.3.3 Native olive oil**	→Hydroxytyrosol and ligstroside share antioxidant and anti-inflammatory properties. These compounds can influence apoptosis and gene regulation, helping to prevent the proliferation of cancer cells. Oleocanthal induces apoptosis in vitro due to increased caspase-3 and polyadenosine diphosphate-ribose polymerase, phosphorylation of p53 (Ser15)] and DNA alteration.
**2.3.4 Native chestnut of the SEAD**	→ Hydrolysable tannins in chestnuts have antioxidant properties, allowing them to neutralize free radicals and protect cells from oxidative damage. Escin and ellagic acid have been shown to have potential anticancer effects by inhibiting the growth of cancer cells and promoting apoptosis.
**2.3.5 Autochthonous honey in the SEAD**	→ Lutein and apigenin inhibited DNMT1, DNMT3a and DNMT3b; inhibited HDACs; induced miR101, protein kinase CK2 (‘casein kinase II’). Kaempferol inhibits death-associated protein kinase 1 (DAPK1) and inhibition of phosphoinositide 3-kinase/protein kinase B signaling pathways. Caffeic acid directly targets protein-serine/threonine kinase ERK1/2 inhibiting them in vitro.
**2.3.6 Wines**	→ Resveratrol targets the AMP-activated protein kinase (AMPK) and the Sirtuin family proteins, the Nuclear factor kappa B (NF-κB) as well as the estrogen receptor (ER). Anthocyanins, flavonols and hydroxicynamic acids have antimetastatic effects
**2.3.7 Milk and dairy derivatives**	→ Some of these peptides have shown antitumor activity or are associated with anticancer properties—lactoferricin, casomorphins, and alpha-lactalbumin
**2.3.8 Dietary Supplementation in the SEAD**	→ There are several studies that support dietary supplementation with olive oil and its benefits to health. Other studies also highlight the anticancer properties of paprika, capsaicinoids, and carotenoids, which are the most characteristic spices of the SEAD. There is widespread interest in dietary supplements containing biomolecules highly present in the SEAD, such as sulforaphane or quercetin.

## Data Availability

Not applicable.
